# Inflammasome Activation Underlying Central Nervous System Deterioration in HIV-Associated Tuberculosis

**DOI:** 10.1093/infdis/jiw561

**Published:** 2016-12-08

**Authors:** Suzaan Marais, Rachel P. J. Lai, Katalin A. Wilkinson, Graeme Meintjes, Anne O’Garra, Robert J. Wilkinson

**Affiliations:** 1 Clinical Infectious Diseases Research Initiative, Institute of Infectious Disease and Molecular Medicine, University of Cape Town, Observatory 7925, South Africa;; 2 Tuberculosis Laboratory and; 3 Laboratory of Immunoregulation and Infection, The Francis Crick Institute,London, and; 4 National Heart and Lung Institute and; 5 Department of Medicine, Imperial College London, United Kingdom

**Keywords:** Tuberculosis meningitis, HIV, microarray, inflammasomes, neutrophils

## Abstract

Tuberculous meningitis (TBM) is a frequent cause of meningitis in individuals with human immunodeficiency virus (HIV) infection, resulting in death in approximately 40% of affected patients. A severe complication of antiretroviral therapy (ART) in these patients is neurological tuberculosis–immune reconstitution inflammatory syndrome (IRIS), but its underlying cause remains poorly understood. To investigate the pathogenesis of TBM-IRIS, we performed longitudinal whole-blood microarray analysis of HIV-infected patients with TBM and reflected the findings at the protein level. Patients in whom TBM-IRIS eventually developed had significantly more abundant neutrophil-associated transcripts, from before development of TBM-IRIS through IRIS symptom onset. After ART initiation, a significantly higher abundance of transcripts associated with canonical and noncanonical inflammasomes was detected in patients with TBM-IRIS than in non-IRIS controls. Whole-blood transcriptome findings complement protein measurement from the site of disease, which together suggest a dominant role for the innate immune system in the pathogenesis of TBM-IRIS.

**(See the editorial commentary by Thuong and Thwaites on pages 665–7.)**

Tuberculosis is the most common cause of death among persons with human immunodeficiency virus (HIV) type 1 infection [1]. The World Health Organization recommends early initiation of antiretroviral therapy (ART) to all patients coinfected with HIV and tuberculosis [2]. Although increased ART coverage of has significantly improved the clinical outcome of patients with HIV-associated tuberculosis, it also leads to an increased incidence of tuberculosis–immune reconstitution inflammatory syndrome (IRIS) [3]. Paradoxical tuberculosis-IRIS is a condition in which patients receiving tuberculosis treatment experience new, recurrent, or worsening of previously stabilized tuberculosis symptoms after commencement of ART. HIV-1–positive patients with tuberculous meningitis (TBM) are at particularly high risk of developing neurological tuberculosis-IRIS (47%). TBM-IRIS is associated with a mortality rate of up to 30% [4–6].

It is well established that serum hypercytokinemia is a major pathological consequence of tuberculosis-IRIS [7]. Similar observations were also reported from cerebrospinal fluid (CSF) in neurologically compartmentalized TBM-IRIS, with significantly higher concentrations of pro- and anti-inflammatory cytokines and chemokines were detected in patients who developed IRIS compared with those who did not [8]. Recently, our group and others have reported that inflammasome activation underlies the immunopathogenesis of tuberculosis-IRIS and that its inhibition results in reduction of cytokine production [9–11]. Inflammasomes are immune complexes of receptors and sensors that mediate innate immune responses and induce inflammation. Canonical inflammasomes are mediated via NOD–like receptor proteins (eg, NLRP3 or NLRC4) or AIM2 and activate caspase 1 to cleave pro–interleukin 1β and pro–interleukin 18 into their mature forms [12]. Noncanonical inflammasomes, on the other hand, activate caspase 4/5 (caspase 11 in mice) to cleave pro–interleukin 1α into its mature form and induce pyroptosis [12]. *Mycobacterium tuberculosis* is known to activate NLRP3 inflammasome and contribute to the tissue-damaging innate inflammatory responses [13, 14].

In this study, we investigated whether inflammasome activation also characterizes the pathogenesis of the highly compartmentalized TBM-IRIS. We performed transcriptomic profiling using microarray on longitudinal whole-blood samples from patients with TBM who did, or did not, develop IRIS after ART. We further performed protein immunoassays on serum and CSF in order to investigate whether TBM-IRIS is associated with a proinflammatory inflammasome-activated innate immune response.

## MATERIALS AND METHODS

### Ethical Approval

The study was approved by the University of Cape Town Human Research Ethics Committee (HREC No. 350/2013). All participants or their relatives provided written informed consent.

### Study Design and Participant Recruitment

Hospitalized patients with HIV-associated TBM were recruited into a prospective observational cohort study at GF Jooste Hospital, a district-level hospital in Cape Town, South Africa, between March 2009 and October 2010 [8, 15]. Eligible patients were adults (aged ≥18 years) with serologically confirmed HIV-1 infection and a diagnosis of TBM according to a published clinical case definition [16]. All patients received antitubercular therapy and prednisone (1.5 mg/kg/d) from the time of TBM presentation and started ART 2 weeks after antitubercular therapy initiation. All *M. tuberculosis* isolates were susceptible to rifampicin and isoniazid, with the exception of 1 isolate from a patient with TBM-IRIS, who was susceptible to rifampicin but not isoniazid. Patients were tracked longitudinally for the development of TBM-IRIS, which was diagnosed according to a published definition [8, 17] (**Figure S1**). Patients who experienced TBM-IRIS received an increased dosage of, or had treated restarted with, prednisone. In patients who did not develop TBM-IRIS, the dosage of prednisone was weaned after 4 weeks of tuberculosis treatment. Another group of HIV-infected, ART-naive adults without tuberculosis and meningitis were enrolled as controls [8].

### Blood and CSF Sample Collection

Paired blood and CSF samples were collected from patients longitudinally at the following time points: TBM presentation, 2 weeks later at the beginning of ART, 2 weeks after ART initiation, and at the time of TBM-IRIS presentation [5]. Plasma and CSF were stored at −80°C until batch RNA analysis and protein determination were performed.

### RNA Extraction and Processing for Microarray Analysis

RNA was extracted from whole blood in PAXgene tubes using PAXgene Blood RNA kits (Qiagen), according to the manufacturer’s protocol. Total RNA (2 μg) was then globin reduced using the GLOBINclear 96-well plate format kit (Thermo Fisher Scientific). The total RNA yield after globin reduction was determined in a NanoDrop 1000 spectrophotometer (Thermo Fisher Scientific), and its integrity was assessed with an Agilent 2100 Bioanalyzer (Agilent Technologies). All RNA samples had an integrity number **>**6.0 and were determined to be acceptable for analysis. Approximately 250 ng of globin-reduced RNA was used to prepare amplified and biotinylated antisense complementary RNA targets, using the Illumina TotalPrep RNA amplification kit (Thermo Fisher Scientific). Labeled complementary RNA (750 ng) was hybridized overnight to Illumina HumanHT-12 v4 BeadChip arrays (Illumina), which contained >47 000 probes (for transcripts). Some genes were represented by >1 probe per transcript. The arrays were then washed, blocked, stained and scanned on an Illumina BeadStation 500. Signal intensity values from the scans were generated using Illumina BeadStudio v2 software. All samples were randomized during all stages of processing to avoid any batch effect. The microarray data from this study have been deposited in the National Center for Biotechnology Information’s Gene Expression Omnibus (accessible through GEO Series accession No. GSE83892).

### Microarray Data Analysis

Raw microarray data were background subtracted, and scaled data were generated using Illumina BeadStudio v2 software. A stringent filter was implemented to select only those transcripts present in 100% of samples from either the IRIS or the non-IRIS group. The transcripts in TBM-IRIS samples were further filtered to have >1.5-fold change in expression compared with the non-IRIS samples. The Mann–Whitney *U* test with the Storey bootstrapping post hoc test was applied with a *Q* value cutoff of <0.05. The transcripts identified were then clustered using a hierarchical algorithm with the Pearson uncentered distance metric and average linkage. The weighted temporal molecular distance was used to determine whether the raw expression value of each differentially expressed transcript in each sample lay within or outside 2 SDs of the baseline control. Mean transcript abundance in patients without meningitis and not receiving ART was used as baseline control. To quantify, transcripts had to differ by ≥200 U in raw signal intensity and ≥2 SDs from the baseline control. The weighted distance is the group mean of the sum of the total SDs for all transcripts meeting these criteria. Functional pathways overrepresented by differentially abundant transcripts were analyzed using Ingenuity Pathway Analysis (Qiagen).

### Quantitative Polymerase Chain Reaction

Total RNA (500 ng) was first reverse-transcribed into complementary DNA using SuperScript VILO Master Mix (Thermo Fisher Scientific) before being used for quantitative polymerase chain reaction. Expression of messenger RNA (mRNA) targets was measured using TaqMan Gene Expression Assays as described elsewhere, and expression of TATA-box binding protein was used as housekeeping [7, 18].

### Serum and CSF Protein Determination

The plasma and CSF concentrations of caspase 1 (R&D Systems), caspase 3 (eBioscience), and caspase 4 (LifeSpan BioSciences) were determined according to manufacturers’ instructions.

### Statistical Analysis

Statistical analyses were performed using GraphPad Prism 6.0 software. Patient baseline characteristics were compared by means of χ^2^ test for contingency data or Kruskal–Wallis test for continuous data. The weighted temporal molecular distances between patients with and those without IRIS were compared using the unpaired *t* test with Welch correction. Potential significance was inferred for results associated with a *P* value <.05.

## RESULTS

### Patient Characteristics

We have previously described the clinical and laboratory findings of our cohort of 34 patients with HIV-associated TBM who did (n = 16) or did not (n = 18) develop paradoxical TBM-IRIS [5, 8]. At time of TBM-IRIS presentation, 15 patients underwent brain imaging, including computed tomography (CT) (n = 14) or magnetic resonance imaging (n = 1). Imaging showed features of TBM in 14 of these patients and normal findings in 1. CT brain abnormalities (excluding generalized atrophy) included extensive focal meningeal enhancement (n = 3), tuberculoma (n = 6), infarcts (n = 4), basal meningeal enhancement (n = 2), and hydrocephalus (n = 2). Of 4 patients in whom CT scans were obtained at TBM diagnosis and at TBM-IRIS presentation, 2 showed worsening radiological features of TBM at TBM-IRIS, including new ring-enhancing lesions (n = 1) and increasing basal meningeal enhancement (n = 1). Serial CT brain findings showed evolving basal ganglia infarcts in the other 2 patients. Magnetic resonance imaging of the spine was performed in 2 of 3 patients with paraparesis, and both had features of radiculomyelitis.

Longitudinal blood samples for RNA analysis were available for 33 of these patients (16 in the TBM-IRIS and 17 in the non-IRIS group) who were included in this study. Seventeen HIV-1–infected patients without tuberculosis or meningitis were included as controls [8]. A tuberculosis diagnosis was excluded in the control patients by lack of tuberculosis symptoms, normal chest radiograph, and absence of tuberculosis treatment. Compared with patients with TBM (TBM-IRIS and non-IRIS), control patients without tuberculosis or meningitis had significantly lower HIV-1 load, CSF lymphocyte and neutrophil counts, and protein concentrations (Table 1). Patients in both TBM-IRIS and non-IRIS groups were similar in terms of sex, age, and baseline CD4 count and HIV-1 load (Table 1). 

**Table 1.** Baseline Characteristics of Patients With HIV-Associated TBM^a^

**Variable**	**Median Value (IQR**)^b^			***P* Value (IRIS vs non-IRIS**)	***P* Value (All TBM vs Controls**)
	**Non-IRIS (n = 17**)	**IRIS (n = 16**)	**Controls (n = 17**)		
Male sex, No. (%)	11 (65)	7 (44)	8 (47)	.23	.62
Age, y	32.0 (25–42)	33.5 (30–46)	34 (26–40)	.43	.54
HIV load, log_10_ copies/mL	5.45 (4.71–5.72)	5.39 (4.75–6.16)	4.85 (3.67–5.46)	.79	.03^c^
Baseline CD4 cell count, cells/mm^3^	103 (77–291)	130 (52–168)	149 (94–354)	.66	.19
CSF values at TBM diagnosis or time of symptom presentation					
Lymphocyte count, 10^6^ cells/L	108 (50–276)	216 (120–419)	7 (3–17)	.06	<.001^c^
Neutrophil count, 10^6^ cells/L	4 (0–33)	38 (11–117)	0 (0–0)	.02^c^	<.001^c^
Protein, g/L	1.38 (1.07–2.13)	2.39 (1.88–4.08)	0.48 (0.37–0.70)	.009^c^	<.001^c^
CSF values 2 wk after ART initiation					
Lymphocyte count, 10^6^ cells/L	26 (5–57)	208 (90–363)	NA	<.001^c^	NA
Neutrophil count, 10^6^ cells/L	0 (0–3)	52 (17–244)	NA	<.001^c^	NA
Protein, g/L	0.61 (0.37–2.04)	3.11 (2.01–22.83)	NA	<.001^c^	NA

Abbreviations: ART, antiretroviral therapy; CSF, cerebrospinal fluid; HIV, human immunodeficiency virus; IQR, interquartile range; IRIS, immune reconstitution inflammatory syndrome; NA, not applicable; TBM, tuberculous meningitis.

^a^Thirty-three patients presented with HIV-associated TBM and were recruited to a prospective cohort. In 17 patients, IRIS did not develop after ART (TBM non-IRIS). In the other 16, TBM-IRIS was diagnosed with a median onset time of 14 days (IQR, 14–20 days) after ART. Sex, age, viral load, and CD4 counts were similar between these 2 groups, but patients with TBM-IRIS had a significantly higher CSF neutrophil counts at baseline (TBM diagnosis) and higher neutrophil and lymphocyte counts 2 weeks after ART initiation.

A control group of 17 HIV-1–infected patients without TBM were also included in the study. Lumbar puncture was performed in the control patients to rule out a central nervous system infection. The final diagnoses in control patients were seizures (n = 4), psychosis (n = 4), nonspecific headaches (n = 4), HIV-associated neurocognitive disorder (n = 2), meningioma (n = 1), stroke (n = 1), and Bell palsy (n = 1). The control and the TBM groups were matched in sex, age, and CD4 cell counts. However, control patients without TBM had a significantly lower HIV-1 loads, CSF lymphocyte and neutrophil counts, and protein concentrations.

^b^Data represent median (IQR) values unless otherwise specified.

^c^Significant at *P* < .05.

All but 1 individual (ie, 15 of 16) in whom TBM-IRIS eventually developed had positive *M. tuberculosis* culture in the CSF at the time of TBM diagnosis, compared with only 6 of 17 patients without IRIS [5]. Time to culture positivity in samples collected during the first study lumbar puncture was similar between patients with TBM-IRIS or without IRIS (median [range], 14 [8–31] vs 15 [4–32] days, respectively). Some patients with TBM-IRIS remained culture positive after 2 weeks (n = 7; median time to culture positivity, 18 days; range, 16–24 days) and 4 weeks (n = 2; time to culture positivity, 16 and 27 days) of tuberculosis treatment. No patients without IRIS were culture positive after starting tuberculosis treatment. In addition, patients with TBM-IRIS also had significantly higher CSF neutrophil counts and CSF protein levels both at the time of TBM diagnosis and 2 weeks after ART initiation (Table 1).

### Changes in Innate Immunity Before the Development of TBM-IRIS

We investigated the transcriptional profile associated with the development of TBM-IRIS using whole-blood microarray, which has been used to characterize non–disease-site-specific tuberculosis-IRIS by our group and others [9, 19]. Whole-blood RNA samples were used owing to the relatively low number of cells present in the CSF compartment, resulting in insufficient amount and quality of RNA available for analysis.

**Figure 1.**
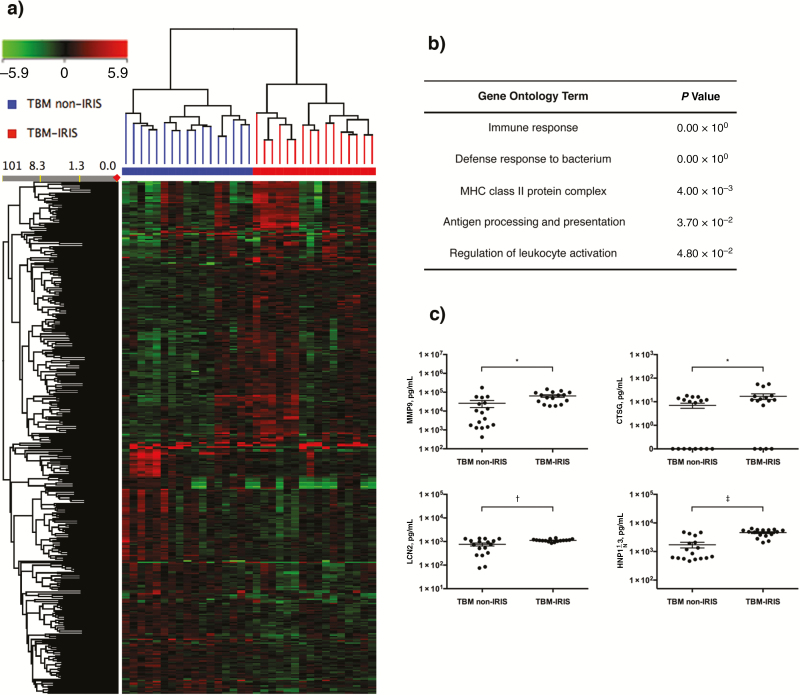
Blood transcriptional signature and cerebrospinal fluid (CSF) protein analysis at the time of tuberculous meningitis (TBM) diagnosis. *A,* Whole-blood samples from 33 patients with human immunodeficiency virus and tuberculosis who had a diagnosis of TBM were processed for microarray. A total of 373 transcripts were found to be differentially abundant (fold change, >1.5) in patients who would later have TBM–immune reconstitution inflammatory syndrome (IRIS) (n = 16), compared with those who would not (TBM non-IRIS; n = 17). On the heat map, up-regulated transcripts appear red, and down-regulated transcripts green. *B,* Gene ontology term analysis on the differentially abundant transcripts showed an increased immune response against *Mycobacterium tuberculosis* pathogen and activation of immune cells in the patients with eventual TBM-IRIS, indicating that they had more active immune responses before beginning any treatment. Abbreviations: MHC, major histocompability complex. *C,* Transcripts (matrix metalloproteinase [MMP] 9, cathepsin G [CTSG], lipocalin 2 [LCN2], and human neutrophil peptide 1-3 [HNP1–3]) associated with neutrophil-dependent inflammatory pathways were significantly up-regulated in the microarray. The CSF protein concentrations of these effectors were significantly higher in the future TBM-IRIS group than in the non-IRIS group. *P < .05; †*P* < .01; ‡*P* < .001 (2-tailed Student *t* tests).

At the time of TBM diagnosis, we identified 373 transcripts that were differentially abundant (fold change [FC], >1.5; *Q* < 0.05) in patients who would eventually develop TBM-IRIS, compared with those who did not (Figure 1A and **Table S1**). Gene ontology term analysis on these 373 transcripts indicated significant overrepresentation of immune response against *M. tuberculosis* bacteria and increased antigen processing and presentation (Figure 1B). We focused particularly on neutrophil-mediated immune responses, because elevated neutrophil counts in the CSF have been implicated as prognostic of future TBM-IRIS development [8]. 

In keeping with the observation in the CSF, the whole-blood transcriptional signature also reflected an increase in neutrophil-dependent inflammatory response with significantly more abundant myeloperoxidase, matrix metalloproteinase (MMP) 8 and 9, cathepsin G (CTSG), lipocalin 2 (LCN2), and α-defensin (DEFA1/3/4) (**Table S1**). To investigate whether the whole-blood transcriptional signature reflected pathology at the disease site, we measured the protein concentrations of these neutrophil mediators in the corresponding CSF samples. Significantly higher protein concentrations of MMP9, CTSG, LCN2, and human neutrophil peptide 1-3 (HNP1-3; encoded by α-defensin 1 and 3 [DEFA1 and DEFA3]) were detected in the CSF of patients who eventually had TBM-IRIS compared with those who did not (Figure 1C). The peripheral transcriptional signature also indicated significant down-regulation of natural killer cell signaling, marked by decreased expression of killer cell immunoglobulinlike receptors 2 and 3 and killer cell lectinlike receptor 2 (**Table S2**).

**Figure 2.**
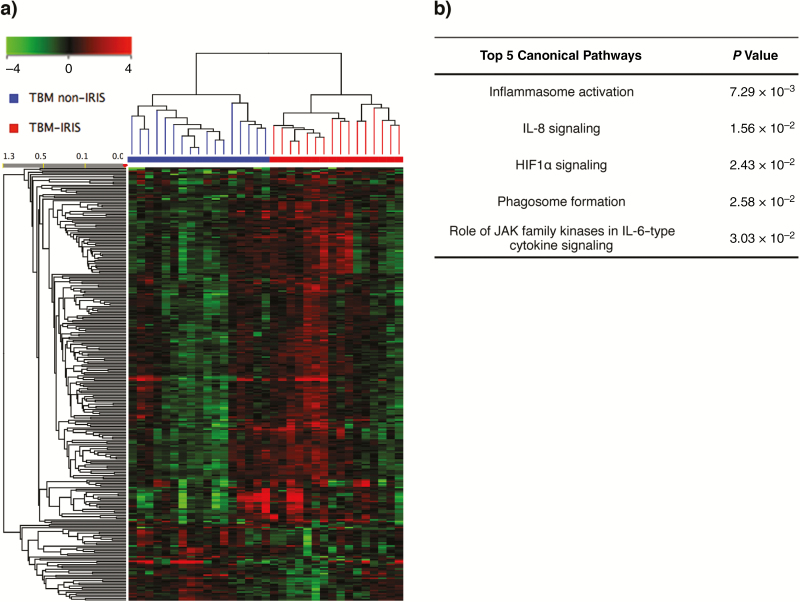
Blood transcriptional signature after antitubercular therapy. Whole-blood samples were taken from patients 2 weeks after antitubercular therapy immediately before starting antiretroviral therapy. *A,* A total of 282 transcripts were found to be differentially abundant (fold change, >1.5) between patients with future tuberculous meningitis (TBM)–immune reconstitution inflammatory syndrome (IRIS) (n = 16) or TBM without IRIS (n = 17). Green color on the heat map represents decreased relative abundance, and red represents increased relative abundance. *B,* Functional analysis identified innate signaling pathways were overrepresented by the 282 transcripts. These include activation of inflammasomes, which have been shown to be mediators of pathogenesis in tuberculosis-IRIS in general. Abbreviations: HIF1α, hypoxia-inducible factor 1-alpha; IL-6, interleukin 6; IL-8, interleukin 8; JAK, Janus kinase.

After the diagnosis of TBM, patients were given antitubercular therapy and prednisone for 2 weeks before ART initiation. Transcriptomic analysis identified 282 differentially abundant (FC, >1.5; *Q* < 0.05) transcripts associated with later development of TBM-IRIS at 2 weeks after tuberculosis treatment and before ART (Figure 2A**andTable S2**). Transcripts associated with neutrophil-dependent inflammation (myeloperoxidase, MMP9 and LCN2) continued to be significantly up-regulated despite the antitubercular and anti-inflammatory (prednisone) therapy (**Table S2**). Further functional analysis of the differentially abundant transcripts also indicated significant up-regulation of several immunological pathways, including inflammasome activation, antigen-presenting cell maturation, and interleukin 8 signaling (Figure 2B), suggesting that patients in whom TBM-IRIS would develop had differences in innate immune response before and after antitubercular therapy and ART.

### TBM-IRIS Onset Associated With Transcripts Involved in Pathogen Recognition and Inflammasome Activation

**Figure 3.**
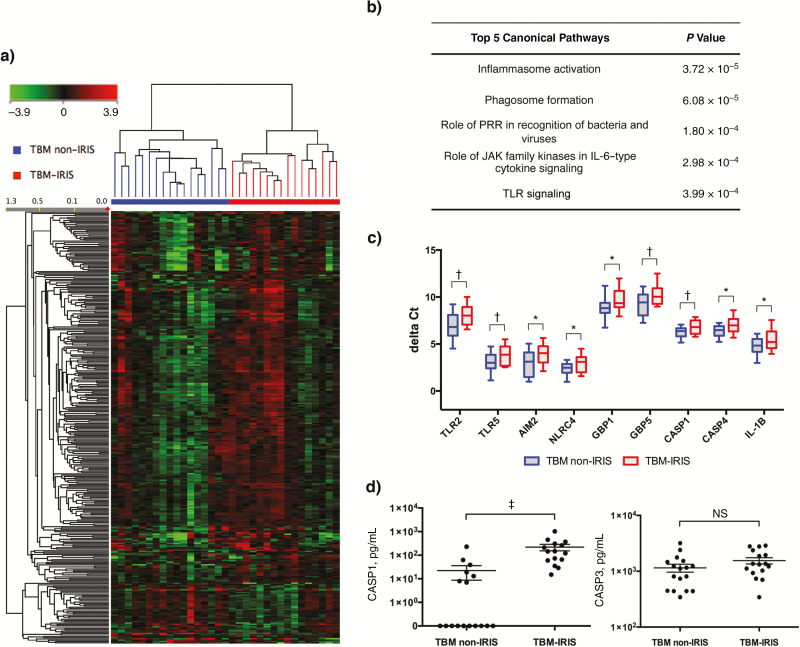
Blood transcriptional signature at the median time of onset for tuberculous meningitis (TBM)–immune reconstitution inflammatory syndrome (IRIS). The median time of IRIS onset was 14 days after antiretroviral therapy (ART) initiation. *A,* A total of 327 transcripts were found to be differentially abundant (fold change, >1.5) in patients with TBM-IRIS (n = 16), compared with those with TBM without IRIS (n = 17). Down-regulated transcripts are shown in green, and up-regulated transcripts are shown in red. *B,* Functional analysis of the transcripts indicated strong involvement of innate immunity at time of symptom onset in TBM-IRIS. These include inflammasome activation mediated by pattern recognition receptors (specifically, Toll-like receptors [TLRs] 1, 2, 6, and 8 and nucleotide-binding oligomerization domain [NOD] 2). Abbreviations: JAK, Janus kinase; PRR, pattern recognition receptor. *C,* Expression of a panel of transcripts associated with inflammasome activation was validated by quantitative polymerase chain reaction. Red represents TBM-IRIS, and blue TBM without IRIS. Elements of both canonical and noncanonical inflammasomes were more abundant in TBM-IRIS at 2 weeks after ART initiation. *P < .05; †*P* < .01 (2-tailed Student *t* tests). AIM2, absence in melanoma 2; CASP, caspase; GBP, guanylate-binding protein; IL-1β, interleukin 1β NLRC4, NOD-like receptor family CARD domain–containing protein 4. *D,* In keeping with the transcriptomic data, the cerebrospinal fluid (CSF) concentration of caspase 1 was significantly higher in patients with TBM-IRIS (‡*P* < .001). The concentrations of CSF caspase 3, which mediates anti-inflammatory apoptosis, did not differ between the TBM non-IRIS and TBM-IRIS groups (not significant [NS]).

In patients in whom TBM-IRIS developed, the median onset time was 14 days (interquartile range, 4–20 days) after ART commencement. We identified 327 differentially abundant (FC, >1.5: *Q* < 0.05) transcripts associated with TBM-IRIS (Figure 3A**andTable S3**) at 2 weeks after ART initiation. Similar to earlier time points, neutrophil-dependent inflammatory responses (MMP9, CTSG, and LCN2) continued to be transcriptionally up-regulated (**Table S3**) in patients with TBM-IRIS. Inflammasome activation has recently been shown as an important mediator of tuberculosis-IRIS in 2 independent studies [9, 11]. In accordance with these results, functional analysis of the differentially abundant transcript also indicated significant up-regulation of transcripts associated with inflammasome activation (Figure 3B). These included signaling via Toll-like receptors and other pattern recognition receptors, such as nucleotide-binding oligomerization domain 2 and C-type lectins, and increased expression of the interferon-induced guanylate-binding proteins 1, 4, 5, and 6 and the noncanonical caspases 4 and 5. 

Elements of the canonical inflammasome, including caspase 1, guanylate-binding protein 2, AIM2, NLRC4, and interleukin 1β, were also up-regulated in TBM-IRIS but with lower FC (1.28–1.48; *Q* < 0.05). The increased mRNA expression of a panel of inflammasome-related genes was validated using quantitative polymerase chain reaction, which confirmed that both canonical and noncanonical inflammasomes were transcriptionally activated in TBM-IRIS (Figure 3C). We further measured the protein concentrations of several caspases in both serum and CSF. Significantly higher amounts of caspase 1 were detected in the CSF, but not serum, of patients with TBM-IRIS compared with those without IRIS (Figure 3D). The concentrations of noncanonical caspase 4 were below the limit of detection in both serum and CSF, whereas the apoptosis-associated caspase 3 did not differ between the 2 groups (Figure 3D). Together, the data suggest that pathogenic mechanism of TBM-IRIS is driven by an aberrant innate immune response, with neutrophils and inflammasome activation being key mediators.

### TBM-IRIS Development Associated With Increased Transcript Perturbation Over Time

**Figure 4.**
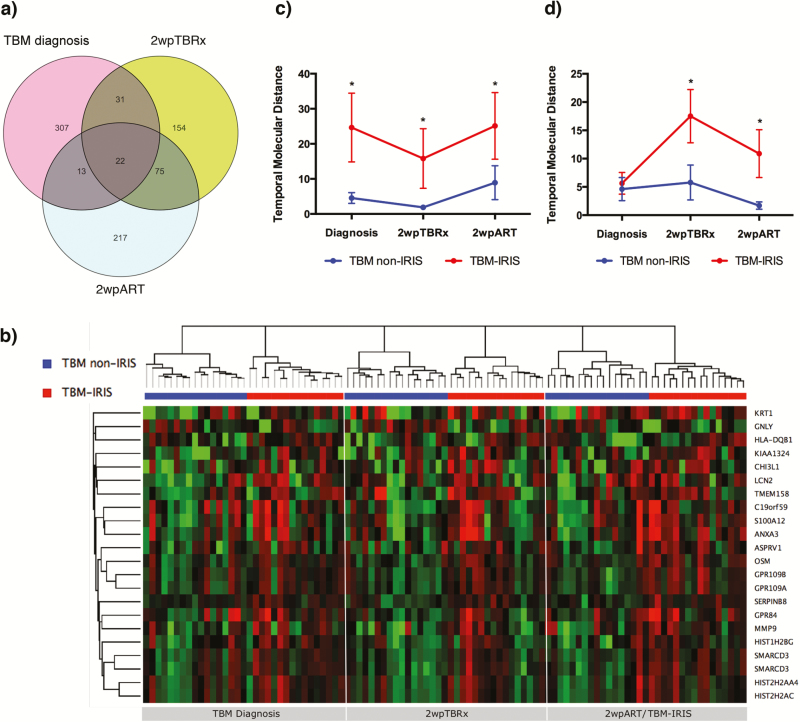
Longitudinal transcriptional perturbation is associated with tuberculous meningitis (TBM)–immune reconstitution inflammatory syndrome (IRIS). *A,* To identify conserved transcriptional features associated with TBM-IRIS development, differentially abundant transcripts at each time point were overlapped: 373 from TBM diagnosis, 282 from 2 weeks after tuberculosis treatment (2wpTBRx), and 327 from 2 weeks after antiretroviral therapy (ART) initiation (2wpART). Twenty-two transcripts (*center*) were found to be consistently differentially abundant in patients with TBM-IRIS over the course of disease development. *B,* Heat map showing the expression of each of the 22 transcripts overtime in patients with TBM without IRIS and patients with TBM-IRIS. Up-regulated transcripts are shown in red, and down-regulated transcripts in green. *C,* Temporal molecular distance, which measures the perturbation of transcript expression, was calculated for the 22 transcripts. The mean expression of these transcripts in human immunodeficiency virus–infected patients without meningitis and not receiving ART (n = 15) was used as the baseline reference. The transcriptional perturbations of these 22 transcripts were significantly higher in the TBM-IRIS than in the TBM non-IRIS group at all 3 time points. *D,* Temporal molecular distance was separately calculated for a list of 21 transcripts associated with inflammasome activation (see Table S4). There was significantly higher transcriptional perturbation in TBM-IRIS at both 2wpTBRx and 2wpART. *P < .05 (2-tailed Student *t* tests). Abbreviations: ANXA3, Annexin A3; ASPRV1, aspartic peptidase retroviral-like 1; C19orf59, mast cell-expressed membrane protein 1; CHI3L1, chitinase-3-like protein 1; GNLY, granulysin; GPR109A, G protein-coupled receptor 109A; GPR109B, G protein-coupled receptor 109B; GRP84, G protein-coupled receptor 84; HIST1H2BG, histone cluster 1 H2B family member G; HIST2H2AA4, histone cluster 2 H2A family member A4; HIST2H2AC, histone cluster 2 H2A family member C; HLA-DQB1, major histocompatibility complex class II, DQ beta 1; KIAA1324, estrogen-induced gene 121; KRT1, keratin 1; LCN2, lipocalin 2; MMP9, matrix metallopeptidase 9; OSM, oncostatin M; S100A12, S100 calcium-binding protein A12; SERPINB8, serpin family B member 8; SMARCD3, SWI/SNF-related matrix-associated actin-dependent; TMEM158, transmembrane protein 158.

We next overlapped the transcript signature identified at each time point (Figure 4A) and found 22 transcripts consistently differentially abundant (21 up-regulated and 1 down-regulated) in patients with TBM-IRIS both before and at the time of symptom onset (Figure 4B). These included the neutrophil-dependent effectors LCN2 and MMP9, proinflammatory mediator oncostatin M and cytolytic protein granulysin. We then applied a weighted temporal molecular distance algorithm [20] to measure the perturbation of these 22 transcripts in each patient sample per time point, relative to the control patients without meningitis. The mean raw intensity valuesof these transcripts from a panel of 15 control patients, who did not have meningitis and were not receiving ART, was used as a baseline control and was given a score of 0. At each of the 3 time points, there were significant perturbations of these 22 transcripts in the patients with TBM-IRIS compared with the non-IRIS samples (Figure 4C). 

Similarly, we also computed the temporal molecular distance for a list of 21 transcripts associated with both canonical and noncanonical inflammasomes that were significantly differentially expressed with an FC of >1.3 (*Q* < 0.05) at 2 weeks after part initiation (**Table S4**). At the time of TBM diagnosis, inflammasome transcript perturbation did not differ between patients who would eventually have TBM-IRIS and those who would not. Whereas the TBM non-IRIS group remained transcriptionally quiescent over time, inflammasome-associated transcripts became significantly more up-regulated in the TBM-IRIS group after antitubercular and antiviral therapies (Figure 4D). Together, these data indicate an early transcriptional increase in proinflammatory mediators and effectors in peripheral blood that precedes the TBM-IRIS episode and suggest that the immunopathological mechanism of IRIS is associated with increased inflammasome activation.

## DISCUSSION

Compared with those who have pulmonary and other forms of extrapulmonary tuberculosis-IRIS, patients with neurological tuberculosis-IRIS often exhibit severe disease, a poorer outcome, and higher mortality rates [6, 21]. To better understand this advanced form of tuberculosis disease, we investigated the transcriptional response in TBM-IRIS, using whole-blood microarray because extraction of disease site-specific RNA was not feasible. Based on past findings in pulmonary tuberculosis [9, 22], we speculate that whole-blood transcriptome would also provide insights on the cellular signaling across the blood-brain-barrier. Furthermore, measuring transcriptional changes in whole blood provides a noninvasive approach to monitor disease prognosis and progress, as an alternative to using CSF samples.

We identified differentially abundant transcripts associated with TBM-IRIS both before and during disease onset, which indicated a key role for neutrophil-dependent and inflammasome-associated proinflammatory responses in the pathogenesis of TBM-IRIS. Previous analyses of CSF from this patient cohort have revealed that increases in bacterial burden, neutrophil counts, and concentrations of the proinflammatory cytokine TNF-α at time of TBM diagnosis predicted future TBM-IRIS [5]. 

In accordance with the CSF data, whole-blood microarray analysis in this study also identified several neutrophil-dependent inflammatory mediators, including MMP9, as having significantly higher transcriptional abundance in patients with TBM-IRIS throughout the course of disease development. MMPs are known to cause lung tissue damage in pulmonary tuberculosis [23]. In the central nervous system, evidence suggests that MMP not only facilitates blood-brain-barrier disruption, which results in leukocyte influx [24, 25], but also up-regulates microglial secretion of cytokines [26]. Furthermore, elevated CSF MMP9 concentrations have been associated with poorer outcome, including death [27] and neurological complications [28, 29], in patients with TBM. In addition to increased CSF concentrations of MMP9 associated with TBM-IRIS, elevated mRNA expression and serum concentrations of MMP9 have also been reported in pulmonary tuberculosis-IRIS [8, 30]. We hypothesize that the consistently high abundance of MMP9 in TBM-IRIS suggests degradation of the extracellular matrix as a mechanism of tissue damage in the central nervous system that is systemically reflected in the blood.

Furthermore, despite having a highly compartmentalized inflammatory response in the CSF, TBM-IRIS seems to share the same underlying pathogenic mechanism with other forms of tuberculosis-IRIS in which inflammasome activation play a key role, and again, this is reflected in the peripheral blood. We have previously shown that Toll-like receptor signaling and inflammasome activation are key mediators of dysregulated cytokine production in a cohort of patients with heterogenous presentations of paradoxical tuberculosis-IRIS [9]. Most recently, Tan and colleagues [11] have also reported that monocytes from patients with tuberculosis-IRIS displayed aberrant inflammasome activation and had greater cell death both before and after ART. 

In keeping with these observations in general tuberculosis-IRIS, we also identified that both canonical and noncanonical inflammasome activation was involved in the pathogenesis of TBM-IRIS. Although there was no discernible difference in the blood transcriptional activity of inflammasomes at the baseline (time of TBM diagnosis), significant transcript perturbations after antitubercular therapy were observed and continued after ART initiation in patients who would go on to have TBM-IRIS. At the site of infection, CSF concentrations of caspase 1 were significantly higher in patients with TBM-IRIS both at TBM diagnosis and also during the IRIS episode, demonstrating that inflammasome activation was both compartmentalized in the central nervous system and reflected systemically at an early stage. Given that inflammasome activation can contribute to pyroptosis [31], an inflammatory cell death distinctive from apoptosis, tissue injury (eg, disruption of the central nervous system architecture and degradation of the extracellular matrix) observed in TBM-IRIS may be partly induced by inflammasome-mediated pyroptosis.

Finally, although patients were prescribed prednisone before any IRIS onset, repression of proinflammatory transcription factors (eg, NF-κB and AP-1) by this anti-inflammatory glucocorticoid [32] was insufficient to prevent the production of inflammatory mediators. Given the unwanted side effects of prednisone, treatments more specific to the pathological mechanism of tuberculosis/TBM-IRIS should be considered. The existence of a small molecule inhibitor (MCC950), which targets the NLRP3 inflammasome [33], suggests more effective ways to both prevent and treat the inflammatory symptoms of tuberculosis-associated IRIS and should be explored in future studies.

## Supplementary Data

Supplementary materials are available at *The Journal of Infectious Diseases* online. Consisting of data provided by the authors to benefit the reader, the posted materials are not copyedited and are the sole responsibility of the authors, so questions or comments should be addressed to the corresponding author.

## Supplementary Material

SupplementaryTableS1

SupplementaryTableS2

SupplementaryTableS3

SupplementaryTableS4

## Notes

***Acknowledgments.*** We thank all the participants in this study and the healthcare workers and administrators at the GF Jooste Hospital. We also thank Nonzwakazi Bangani for technical assistance.

***Author contributions.*** S. M., R. P. J. L., G. M., A. O., and R. J. W. conceived and designed the study. S. M. recruited, sampled and collected data from patients. S. M., R. P. J. L., and K. A. W. performed the experiments. S. M. and R. P. J. L. analyzed the data. S. M., R. P. J. L., and R. J. W. wrote the manuscript, which was revised by K. A. W., G. M., and A. O.

***Disclaimer.*** The funders had no role in the study design, data collection, data analysis, data interpretation, or writing of this report. The opinions, findings and conclusions expressed in this manuscript reflect those of the authors alone.

***Financial support.***  This work was supported by the Carnegie Corporation Training Award and Discovery Foundation Academic Fellowship Award (S. M.); the Perinatal HIV Research Unit, US Agency for International Development, and the President’s Emergency Plan for AIDS Relief (S. M.); Wellcome Trust (grants WT 097254, 081667, 084323, 097684, and 104803 to S.M., G. M., and R. J. W. and 098316 to G. M.); the Fogarty International Center, National Institutes of Health (grants U2R TW007373-01A1 and U2R TW007370-01A1); the National Research Foundation of South Africa (grant 96841 to R. J. W. and incentive funding UID 85858 to G. M.); The Francis Crick Institute (grant 10126 to A. O. and 10218 to R. J. W.); the UK Medical Research Council, Wellcome Trust and Cancer Research UK (funding to The Francis Crick Institute); the European & Developing Countries Clinical Trials Partnership (grant SP.2011.41304.074 to G. M.); the South African Research Chairs Initiative of the Department of Science and Technology and National Research Foundation of South Africa (grant 64787 to G. M.); and the South African Medical Research Council through its TB and HIV Collaborating Centres Programme with funds received from the National Department of Health (RFA SAMRC-RFA-CC: TB/HIV/AIDS-01-2014 to G. M.).

***Potential conflicts of interest.*** All authors: No reported conflicts. All authors have submitted the ICMJE Form for Disclosure of Potential Conflicts of Interest. Conflicts that the editors consider relevant to the content of the manuscript have been disclosed.
