# Supplementary material for: Inflammasome Activation Underlying Central Nervous System Deterioration in HIV-Associated Tuberculosis
Source: J Infect Dis. 2016 Dec 8;215(5):677–86. doi: 10.1093/infdis/jiw561 (PMC5388298; doi:10.1093/infdis/jiw561)
Supplement: SupplementaryTableS1 [file jiw561_suppl_SupplementaryTableS1.pdf]

Illumina_ProbeID	Symbol	Regulation	FC	p -value	q-value
5050598	LOC645381	down	-2.07	1.19E-07	7.52E-08
1570086	B3GNTL1	up	1.51	9.27E-03	5.28E-05
6100356	ALPL	up	1.69	8.39E-02	1.65E-04
2070646	GPR84	up	1.69	6.02E-02	1.28E-04
620136	LILRA5	up	1.53	3.64E-02	9.50E-05
5860075	CAMP	up	1.72	9.78E-02	1.87E-04
2320152	ASB16	down	-1.73	2.94E-02	8.54E-05
4290731	KREMEN1	up	1.76	3.40E-02	9.21E-05
3130370	ZNF83	up	1.51	1.99E-02	7.28E-05
4590519	LOC100130332	up	1.55	1.30E-02	6.13E-05
450020	ORM2	up	1.54	2.28E-02	7.55E-05
3800600	MAGED1	down	-1.59	6.53E-03	4.79E-05
4540475	FABP5L2	down	-1.52	1.27E-02	6.03E-05
3830164	C14orf145	down	-2.01	3.25E-05	1.03E-05
6580041	GNLY	down	-1.50	1.20E-01	2.15E-04
580403	DFNA5	down	-1.64	4.29E-02	1.04E-04
5260484	HLA-DRB1	down	-6.38	3.92E-03	4.74E-05
6480131	ADAM15	up	1.58	1.24E-03	3.66E-05
3180220	GPR109B	up	1.52	3.64E-02	9.50E-05
4860255	ASPRV1	up	1.54	1.08E-01	1.99E-04
3390097	LOC728093	up	1.52	7.57E-02	1.52E-04
380102	TMEM51	down	-1.53	5.48E-02	1.23E-04
1010528	HES1	down	-1.52	1.14E-02	5.74E-05
2760500	CD38	down	-1.70	8.89E-03	5.24E-05
4200669	CR1	up	1.58	5.69E-02	1.24E-04
60674	LOC652102	down	-1.83	3.98E-02	9.94E-05
270136	RFX2	up	1.93	1.24E-02	5.99E-05
6550632	ZNF219	up	1.62	6.03E-03	4.74E-05
3390424	ITGAD	down	-1.77	5.05E-03	4.74E-05
1400474	MYO1D	down	-1.72	5.03E-03	4.74E-05
3850398	STX2	up	1.54	1.51E-02	6.54E-05
1090307	RNASE1	up	1.56	5.54E-02	1.23E-04
4920612	GNLY	down	-1.64	9.16E-02	1.77E-04
4060278	PLGLA	down	-1.60	3.13E-02	8.77E-05
3800021	LOC647506	down	-1.76	1.98E-02	7.28E-05
1450608	VWCE	up	1.72	1.83E-01	3.16E-04
1850687	PRKY	down	-2.96	4.00E-03	4.74E-05
2230678	ACACB	down	-1.53	4.39E-02	1.05E-04
6200196	SLC4A8	down	-1.61	4.86E-04	2.56E-05
2450270	PSORS1C1	down	-1.77	1.74E-02	6.92E-05
1990706	LOC284648	up	2.23	4.13E-03	4.74E-05
380440	NA	up	1.58	3.22E-03	4.74E-05
1570743	METTL9	up	1.57	1.99E-02	7.28E-05
3830228	GPR109A	up	1.56	2.89E-02	8.51E-05
5310445	KREMEN1	up	1.56	1.33E-01	2.35E-04
3390301	KREMEN1	up	1.79	7.20E-02	1.47E-04
5310754	VNN1	up	1.57	1.52E-01	2.66E-04
6590224	WFS1	down	-1.68	6.25E-03	4.74E-05
7560170	LOC441864	up	1.64	1.48E-02	6.51E-05
6550164	DEFA4	up	3.09	1.96E-02	7.28E-05
510072	LOC100132317	up	1.54	7.09E-02	1.46E-04
4050376	GYPE	up	1.87	9.34E-04	3.30E-05
4640500	UCHL1	down	-1.54	5.93E-02	1.28E-04
2140719	KIR2DL4	down	-1.82	2.21E-02	7.42E-05
5310154	GRIP2	up	1.53	3.04E-02	8.58E-05
1260228	CLEC5A	up	2.40	5.93E-03	4.74E-05
6980192	HBZ	down	-2.50	7.26E-02	1.47E-04
1990037	CHI3L1	up	1.79	1.08E-01	1.99E-04
1580437	PGA5	up	1.55	4.65E-02	1.09E-04
2630711	MCM4	down	-1.56	3.63E-02	9.50E-05
6370315	HLA-DRB5	down	-1.91	3.34E-01	5.68E-04
6900241	ABCB9	down	-1.91	7.12E-04	2.99E-05
5290598	ABCB9	down	-2.17	7.16E-05	1.13E-05
3170068	APOBEC3B	down	-1.67	2.13E-02	7.40E-05
2070224	BUB1	down	-1.65	5.79E-02	1.25E-04
5080692	HLA-A29.1	up	2.08	2.41E-01	4.14E-04
3710553	SMOX	up	1.63	1.41E-01	2.49E-04
2600121	OR4K15	up	1.80	1.53E-02	6.57E-05
4390398	LCN2	up	2.37	1.87E-02	7.21E-05
7050201	NA	down	-2.25	1.23E-02	5.99E-05
6840301	NT5DC2	down	-1.50	5.52E-02	1.23E-04
7650497	ELANE	up	2.92	1.87E-02	7.21E-05
7150609	ADAMTS2	up	1.77	3.58E-03	4.74E-05
520360	MS4A6A	up	1.52	5.17E-03	4.74E-05
6330184	HBBP1	up	1.53	8.06E-02	1.59E-04
6590131	HP	up	2.06	1.13E-01	2.06E-04
430328	LOC100130520	down	-1.81	4.01E-02	9.97E-05
1820747	DAAM2	up	1.67	1.27E-02	6.03E-05
7040161	HELLS	down	-1.66	2.66E-03	4.58E-05
3780100	OSBP2	up	1.62	2.67E-01	4.56E-04
2100458	LOC729010	up	1.51	3.52E-02	9.49E-05
3360615	FCER1A	down	-1.81	7.27E-02	1.47E-04
4850168	SLC22A4	up	1.54	4.19E-02	1.03E-04
2850315	ORM1	up	2.49	2.86E-02	8.51E-05
6040398	SLC22A4	up	1.55	4.47E-02	1.06E-04
2710452	POU2AF1	down	-1.67	6.90E-03	4.82E-05
5270753	ARG1	up	1.94	7.61E-02	1.52E-04
2570497	LOC100134379	up	2.11	2.30E-02	7.55E-05
5420066	H6PD	up	1.56	1.06E-02	5.64E-05
940673	PACAP	down	-1.62	3.84E-02	9.82E-05
2370072	PRR11	down	-1.51	9.26E-03	5.28E-05
6650333	LOC653867	up	1.64	1.64E-02	6.84E-05
7380273	GPR114	down	-1.58	7.43E-03	4.94E-05
1190528	MANSC1	up	1.52	1.18E-01	2.13E-04
3840561	UTY	down	-1.56	1.32E-01	2.34E-04
270068	SMOX	up	1.62	1.32E-01	2.35E-04
6580072	SMOX	up	1.82	7.49E-02	1.51E-04
1430484	C21orf81	down	-1.69	4.99E-02	1.15E-04
1500296	LOC283392	up	2.05	6.92E-03	4.82E-05
780079	LOC654053	down	-1.67	1.18E-01	2.13E-04
6560079	SUCNR1	up	1.73	2.30E-02	7.55E-05
6250010	GPRIN3	down	-1.62	1.67E-02	6.90E-05
1780537	ABCA13	up	1.59	1.87E-02	7.21E-05
650689	CLIP3	down	-1.52	4.66E-03	4.74E-05
4890181	RAP1GAP	up	1.77	3.88E-01	6.58E-04
3460594	C14orf68	down	-1.69	2.40E-02	7.67E-05
2710142	IER5L	up	1.56	5.36E-03	4.74E-05
6580482	ADORA3	down	-2.29	6.25E-03	4.74E-05
5960132	NOLC1	down	-1.53	1.10E-02	5.66E-05
3400762	LOC643870	up	1.67	3.67E-02	9.52E-05
4180524	EZH2	down	-1.58	1.38E-02	6.29E-05
7150630	HLA-C	down	-2.24	1.63E-01	2.84E-04
4290133	PACSIN1	down	-1.60	1.27E-02	6.03E-05
6980592	NA	up	1.51	8.66E-03	5.20E-05
3780673	EMR4	down	-1.55	5.19E-02	1.19E-04
4220110	LOC647450	down	-1.57	2.98E-02	8.54E-05
3520102	CCL4L1	down	-1.56	4.98E-03	4.74E-05
4050717	MYOM2	down	-2.72	4.10E-03	4.74E-05
5550367	LTF	up	1.86	6.02E-02	1.28E-04
6450102	LOC642113	down	-1.53	5.08E-02	1.16E-04
2000128	C4BPA	up	3.68	4.11E-03	4.74E-05
2450544	IL5RA	down	-1.83	9.29E-03	5.28E-05
6280594	MMP8	up	3.55	5.61E-03	4.74E-05
6370474	CDCA3	down	-1.59	2.83E-02	8.51E-05
5390427	NAIP	up	1.67	2.20E-02	7.42E-05
5340240	NAIP	up	1.59	4.29E-02	1.04E-04
5290100	MAK	up	1.57	4.48E-02	1.06E-04

2190671	CCR3	down	-1.97	2.56E-02	8.01E-05
6860706	LOC729891	up	1.51	2.69E-02	8.24E-05
7320324	EMR4P	down	-1.85	3.93E-02	9.90E-05
1510735	NTN3	up	1.69	1.05E-01	1.97E-04
2320441	LOC23117	down	-1.53	3.94E-02	9.90E-05
150609	LOC652493	down	-1.66	1.43E-02	6.37E-05
1580360	LOC651612	up	1.74	4.38E-02	1.05E-04
2570605	ARHGAP5	up	1.63	8.65E-03	5.20E-05
5550019	GPRC5D	down	-1.66	2.01E-02	7.28E-05
6560408	NA	up	1.53	3.27E-03	4.74E-05
3390253	TMEM156	down	-1.55	1.10E-02	5.66E-05
3870497	FUT8	down	-1.54	1.37E-02	6.29E-05
1400348	NMUR1	down	-1.58	1.56E-02	6.59E-05
4890750	DDX11	down	-1.78	2.90E-03	4.74E-05
7380047	THRA	up	1.51	4.29E-03	4.74E-05
3840113	PRDM1	down	-1.61	1.91E-03	4.09E-05
5260278	PPAPDC3	up	1.73	3.55E-02	9.49E-05
2760678	RCVRN	up	1.58	1.05E-02	5.64E-05
5870739	JARID1D	down	-2.80	7.75E-03	4.94E-05
2450647	KRT1	up	1.71	2.46E-01	4.22E-04
7210632	AKR1C3	down	-1.52	9.41E-02	1.82E-04
1400093	LOC401845	down	-1.63	3.99E-02	9.94E-05
4210397	GLDC	down	-1.82	2.35E-02	7.67E-05
7160474	HLA-DQB1	up	2.44	6.42E-03	4.76E-05
5390497	C7orf53	up	1.75	2.58E-02	8.02E-05
6280086	LGALS2	down	-1.96	4.14E-03	4.74E-05
5890653	CDKN1C	down	-2.51	4.35E-04	2.49E-05
4780619	AKAP7	down	-1.55	1.45E-01	2.54E-04
2350546	GPR55	down	-1.59	3.01E-02	8.54E-05
5560400	SLC14A1	up	1.74	5.73E-02	1.25E-04
2690181	NECAB2	up	1.54	1.27E-01	2.28E-04
5670739	AZU1	up	2.77	2.02E-02	7.29E-05
5260008	C1orf183	up	1.59	5.76E-02	1.25E-04
1340670	KIR2DL3	down	-1.80	9.99E-03	5.46E-05
3140242	KIR2DL3	down	-1.74	6.31E-03	4.74E-05
1070367	C19orf59	up	1.76	1.01E-01	1.90E-04
6520593	RNF182	up	1.95	1.12E-01	2.05E-04
780035	SYTL2	down	-1.58	5.95E-03	4.74E-05
4180647	SHPRH	down	-1.54	1.47E-03	4.03E-05
1940368	NRSN2	down	-1.83	3.41E-03	4.74E-05
7050639	LOC100130520	down	-1.74	6.63E-02	1.39E-04
1190446	DLC1	down	-1.61	4.07E-01	6.89E-04
2100725	KIR3DL3	down	-1.50	1.16E-01	2.12E-04
2810692	KIAA1881	up	1.54	1.11E-01	2.05E-04
6380411	IKZF3	down	-2.12	2.94E-02	8.54E-05
5050148	CDRT4	down	-1.56	3.63E-03	4.74E-05
780504	LOC442535	down	-2.17	6.73E-03	4.82E-05
270114	FAM134B	up	1.91	7.53E-03	4.94E-05
1410730	VSIG4	up	1.68	9.02E-02	1.76E-04
4610543	TLE1	down	-1.61	2.01E-03	4.09E-05
2850471	KCTD12	up	1.96	1.05E-01	1.97E-04
6960553	TERF1	down	-1.57	5.96E-05	1.13E-05
6100687	RPS4Y1	down	-3.55	2.00E-02	7.28E-05
940747	CAMK1G	down	-1.50	4.53E-02	1.07E-04
4570451	LOC401233	up	2.41	1.83E-03	4.09E-05
6650193	PTPRC	down	-1.51	7.27E-02	1.47E-04
2030678	HIST2H2AB	up	1.71	8.19E-02	1.61E-04
1470673	RFX2	up	1.80	3.55E-02	9.49E-05
2680189	LAG3	down	-1.52	4.30E-02	1.04E-04
6590575	SPRY2	down	-1.58	7.54E-03	4.94E-05
2760307	NA	down	-1.87	2.37E-03	4.58E-05
1070215	CAV1	down	-1.67	4.45E-03	4.74E-05
6330440	MSH5	up	1.53	3.86E-03	4.74E-05
7380452	KLHDC8A	up	1.51	1.73E-01	3.00E-04
5910632	SMARCD3	up	1.52	2.87E-02	8.51E-05
6100484	TNNI2	up	1.79	3.32E-04	2.49E-05
5340201	LOC100132535	down	-1.84	1.86E-03	4.09E-05
2070561	KIR2DS5	down	-1.54	3.40E-02	9.21E-05
540647	TMEM56	up	1.65	7.67E-02	1.53E-04
7400377	CEACAM6	up	3.04	1.70E-02	6.92E-05
1690440	XIST	up	1.73	3.19E-02	8.89E-05
2000669	ARHGAP10	down	-1.60	5.70E-03	4.74E-05
5080180	CACNG6	down	-1.99	1.98E-02	7.28E-05
6480468	BEX1	up	1.72	6.76E-02	1.40E-04
5360048	CCL23	down	-2.86	3.15E-03	4.74E-05
7040735	CYP27A1	up	1.72	5.37E-02	1.21E-04
4050156	ERBB2	down	-1.50	2.72E-02	8.25E-05
3440327	LOC653061	up	2.49	5.95E-03	4.74E-05
3780647	KIAA0125	down	-1.53	1.77E-02	6.96E-05
290669	SLC1A7	down	-1.60	2.63E-02	8.09E-05
6110343	CCL23	down	-3.41	1.16E-02	5.76E-05
2320358	SLC39A8	down	-1.56	6.72E-02	1.40E-04
6370646	CEACAM4	up	1.50	7.96E-02	1.58E-04
2900360	KIR2DL4	down	-1.79	3.15E-03	4.74E-05
4920075	TGM3	up	1.74	2.85E-02	8.51E-05
4290148	HIST2H2AA4	up	1.53	5.35E-02	1.21E-04
5420725	PNPLA1	up	1.55	4.90E-02	1.14E-04
3130220	TMEM158	up	1.61	1.06E-01	1.98E-04
450609	IGLL3	down	-1.94	9.12E-04	3.30E-05
1500735	CTSG	up	2.13	1.15E-01	2.10E-04
4640553	KIR3DL1	down	-1.59	3.40E-02	9.21E-05
2360164	KLRC2	down	-1.78	3.38E-02	9.21E-05
4830541	HBE1	up	1.73	2.21E-01	3.80E-04
3400672	SERPINB8	up	1.52	1.17E-03	3.66E-05
4260338	LOC647307	down	-1.52	9.74E-03	5.39E-05
7650736	NA	up	1.62	2.43E-03	4.58E-05
5570711	IDO1	down	-1.91	9.97E-02	1.89E-04
6280646	LOC100133662	down	-2.87	2.00E-02	7.28E-05
6580626	CXorf57	down	-1.53	7.97E-03	5.03E-05
620484	TNFRSF13B	down	-1.75	1.37E-02	6.29E-05
1050008	MMRN1	up	1.74	1.08E-01	1.99E-04
110397	LOC652755	up	1.62	6.08E-03	4.74E-05
4540239	DEFA1	up	1.95	6.34E-02	1.33E-04
7400402	HIST1H4D	up	1.52	9.77E-02	1.87E-04
7000546	LILRA6	up	1.81	5.42E-03	4.74E-05
6180743	LAIR2	down	-2.12	2.18E-02	7.42E-05
6020037	JSRP1	down	-1.64	2.59E-02	8.02E-05
2260349	MIR1974	up	1.51	5.36E-02	1.21E-04
4200746	BPI	up	1.89	8.59E-02	1.68E-04
4570612	FASLG	down	-1.51	3.27E-02	9.08E-05
2120634	CMTM4	up	1.51	2.08E-02	7.33E-05
1740328	KIR2DL5A	down	-1.61	5.25E-03	4.74E-05
830615	ZDHHC19	up	2.03	1.57E-01	2.74E-04
3170066	FANCI	down	-1.51	2.70E-02	8.24E-05
2360356	FSTL3	up	2.07	3.88E-04	2.49E-05
4290026	C20orf103	down	-2.40	2.04E-04	1.95E-05
6290471	LOC100134424	up	1.78	2.16E-04	1.95E-05
2490463	MXI1	up	1.56	2.10E-01	3.62E-04
1850523	GZMB	down	-1.65	4.94E-03	4.74E-05
1780239	OLIG2	down	-2.02	4.65E-02	1.09E-04
940670	TRAM2	down	-1.58	1.60E-02	6.74E-05
6290328	MAFG	up	1.53	5.80E-04	2.81E-05
1050360	HLA-DPB1	down	-1.51	4.08E-02	1.01E-04
870497	CCNE1	down	-1.50	2.06E-02	7.31E-05
4570220	LOC100130422	down	-1.54	2.11E-02	7.40E-05
6270138	TACSTD2	up	2.39	1.47E-02	6.51E-05
7610348	LOC652775	down	-1.68	2.27E-02	7.55E-05
6450619	SPON2	down	-1.86	1.00E-02	5.46E-05

380259	INDO	down	-2.10	7.16E-02	1.47E-04
6250615	PGLYRP1	up	1.63	1.06E-01	1.98E-04
3850647	PTPN20	up	2.73	1.92E-03	4.09E-05
7150170	DEFA1B	up	2.82	2.21E-02	7.42E-05
870477	DEFA1B	up	2.52	2.98E-02	8.54E-05
4860128	DEFA1B	up	2.29	4.42E-02	1.06E-04
4010296	RNASE1	up	1.71	9.87E-02	1.87E-04
6350437	CELSR3	down	-1.55	7.66E-03	4.94E-05
5050180	LOC652113	down	-2.03	1.98E-03	4.09E-05
6900523	EMR4	down	-1.81	4.49E-02	1.06E-04
5870474	RHOBTB3	down	-2.08	1.06E-03	3.52E-05
6100022	HIST2H2AC	up	1.51	5.63E-02	1.24E-04
1190754	LOC650557	down	-2.13	1.65E-02	6.84E-05
4610129	RETN	up	3.05	1.39E-02	6.29E-05
1770731	PRTN3	up	2.40	1.72E-02	6.92E-05
940519	GPR44	down	-2.44	8.85E-03	5.24E-05
1500725	CD1C	down	-1.57	2.58E-03	4.58E-05
770025	SPDYE1	up	2.23	1.28E-03	3.66E-05
540477	KIF19	down	-1.56	1.55E-02	6.59E-05
620301	TECPR2	up	1.53	4.13E-02	1.01E-04
2970747	DEFA3	up	2.29	4.85E-02	1.13E-04
6860102	CYorf15A	down	-2.23	4.31E-03	4.74E-05
2340743	CPA3	down	-1.73	3.37E-02	9.21E-05
3840072	PRG2	up	1.80	1.06E-02	5.64E-05
780538	ANRIL	up	1.54	2.93E-02	8.54E-05
580445	LOC649923	down	-1.57	1.40E-01	2.48E-04
7510132	OLR1	up	2.59	8.19E-03	5.06E-05
7570102	CYorf15B	down	-1.51	7.23E-03	4.94E-05
3830762	TMEM119	up	2.51	3.60E-02	9.50E-05
6770131	OLFM4	up	4.73	2.69E-03	4.58E-05
5560471	FCAR	up	1.53	6.02E-02	1.28E-04
130743	CLEC4C	up	1.52	1.99E-02	7.28E-05
7650008	NLRP12	up	1.68	6.18E-03	4.74E-05
430546	HIST1H2BG	up	1.73	1.75E-02	6.92E-05
1850402	LOC731007	down	-1.56	2.38E-02	7.67E-05
1580681	MS4A3	up	2.05	2.15E-02	7.40E-05
6290228	UTS2	up	1.62	5.80E-03	4.74E-05
6180687	SLC29A1	down	-1.81	1.99E-02	7.28E-05
5910424	SLC29A1	down	-2.27	7.55E-03	4.94E-05
2470364	PRSS33	down	-2.56	2.46E-02	7.81E-05
1770603	TCN1	up	2.00	2.57E-02	8.01E-05
3180528	MMP9	up	1.57	1.20E-01	2.16E-04
2760463	PKD2L1	up	1.52	2.04E-02	7.29E-05
460463	SMARCD3	up	1.68	1.10E-02	5.66E-05
460113	MAGED1	down	-1.52	6.67E-03	4.82E-05
770026	TMEM38A	up	1.55	1.11E-02	5.66E-05
4880553	MFSD9	up	1.51	7.74E-03	4.94E-05
5050333	RHAG	up	1.66	7.81E-02	1.55E-04
1070463	CYorf15B	down	-2.12	3.45E-03	4.74E-05
20075	NDUFAF3	up	1.52	9.81E-02	1.87E-04
4220692	CENTG2	down	-1.86	2.55E-03	4.58E-05
3870170	LOC728093	up	1.53	4.04E-02	1.00E-04
5050053	TXNDC5	down	-1.60	1.41E-02	6.34E-05
7330341	TXNDC5	down	-1.79	6.78E-02	1.40E-04
4560746	FCAR	up	1.55	3.92E-02	9.90E-05
1230615	WFDC1	up	1.67	2.16E-02	7.41E-05
4850398	TXNDC5	down	-1.90	1.10E-02	5.66E-05
2750706	NTSR1	up	2.05	6.17E-03	4.74E-05
770400	LOC653600	up	3.55	2.39E-02	7.67E-05
2810673	HOXA9	up	1.52	3.85E-02	9.82E-05
50278	LAIR2	down	-2.07	2.87E-02	8.51E-05
1010736	TOX2	down	-1.64	5.26E-03	4.74E-05
10333	LOC731682	up	2.64	5.70E-02	1.24E-04
5390168	SH2D1B	down	-1.74	5.54E-03	4.74E-05
3520601	MPO	up	1.96	6.08E-02	1.29E-04
580136	LOC652694	down	-1.91	1.71E-02	6.92E-05
1940632	NCAPG2	down	-1.54	1.74E-02	6.92E-05
1230524	ST6GALNAC3	up	1.51	1.33E-02	6.22E-05
1410221	S100A12	up	1.73	9.83E-02	1.87E-04
5050544	MS4A3	up	1.79	9.03E-02	1.76E-04
7200025	KIR2DL1	down	-1.67	9.05E-02	1.76E-04
4210458	KIR2DL1	down	-1.63	3.57E-02	9.49E-05
2230601	UTS2	up	1.58	2.47E-02	7.81E-05
6860048	UTS2	up	1.57	8.08E-03	5.05E-05
5890095	LILRA5	up	1.57	2.54E-02	8.01E-05
7100274	ALDH6A1	down	-1.77	6.54E-04	2.95E-05
4590551	TRA1P2	down	-1.83	1.99E-03	4.09E-05
770682	MGC29506	down	-1.67	1.86E-02	7.21E-05
2030142	CLC	down	-1.97	5.52E-02	1.23E-04
5290079	GLCCI1	down	-1.83	9.56E-05	1.21E-05
240594	ANXA3	up	1.62	1.03E-01	1.95E-04
3800132	TMPRSS9	down	-2.15	5.38E-02	1.21E-04
7330398	HLA-DRB4	up	2.09	2.78E-01	4.74E-04
4570398	F2R	down	-1.50	5.50E-03	4.74E-05
4780075	CEACAM8	up	2.61	2.95E-02	8.54E-05
3060767	MANEA	down	-1.64	1.51E-02	6.54E-05
5860138	RIPK4	up	1.63	4.04E-04	2.49E-05
4150600	EIF1AY	down	-2.48	6.04E-02	1.28E-04
20397	ALOX15	down	-1.85	3.77E-02	9.74E-05
1230722	ALOX15	down	-1.63	6.27E-02	1.32E-04
6280326	HDC	down	-1.81	3.36E-02	9.21E-05
1240554	TNFRSF17	down	-1.72	5.32E-02	1.21E-04
5900170	SLC11A1	up	1.53	1.19E-02	5.85E-05
2900669	RAB30	down	-1.82	3.12E-03	4.74E-05
7560593	OSM	up	1.51	3.09E-02	8.71E-05
4250059	HRASLS2	down	-1.52	2.05E-02	7.31E-05
2260189	FLJ14166	up	1.50	3.89E-02	9.89E-05
3780370	C9orf72	up	1.59	9.14E-03	5.28E-05
840253	ALDH2	up	1.55	1.50E-02	6.54E-05
990372	COL17A1	up	2.53	9.65E-03	5.38E-05
2120270	COL17A1	up	1.79	4.10E-03	4.74E-05
3170241	LOC651524	up	1.96	8.37E-03	5.13E-05
4210039	ABCB1	down	-1.50	2.15E-02	7.40E-05
520609	KIAA1324	up	2.52	6.95E-03	4.82E-05
2650220	FSTL1	up	1.69	6.64E-02	1.39E-04
4230102	SOCS3	up	1.57	5.62E-02	1.24E-04
3180719	FLJ10916	up	1.53	6.87E-02	1.42E-04
2030403	OLIG1	down	-1.64	1.25E-01	2.24E-04
6020273	RPS4Y2	down	-1.70	3.84E-02	9.82E-05
2640671	LOC642342	up	1.65	3.00E-02	8.54E-05
2680605	IGJ	down	-1.91	4.92E-03	4.74E-05
4040176	LAMA5	down	-1.52	2.37E-02	7.67E-05
5960068	PDGFRB	down	-1.93	9.51E-03	5.35E-05
10451	BEND7	up	1.52	4.97E-02	1.15E-04
1710673	LOC652126	down	-1.70	3.62E-02	9.50E-05
6420309	FAM134B	up	2.05	1.89E-03	4.09E-05
3180661	FAM134B	up	1.81	1.16E-02	5.76E-05
2480280	SORD	down	-1.70	9.41E-04	3.30E-05
