# Supplementary material for: Inflammasome Activation Underlying Central Nervous System Deterioration in HIV-Associated Tuberculosis
Source: J Infect Dis. 2016 Dec 8;215(5):677–86. doi: 10.1093/infdis/jiw561 (PMC5388298; doi:10.1093/infdis/jiw561)
Supplement: SupplementaryTableS2 [file jiw561_suppl_SupplementaryTableS2.pdf]

Illumina_ProbeID	Symbol	Regulation	FC	p-value	q-value
770639	PDLIM7	up	1.50	3.39E-02	1.30E-04
2190349	KCNJ15	up	2.22	3.19E-03	8.33E-05
5570139	QPCT	up	1.62	1.84E-02	9.87E-05
540288	TMEM88	up	1.55	5.79E-02	1.73E-04
6550333	CXCR6	down	-1.57	2.59E-02	1.11E-04
3190189	TTRAP	up	1.52	4.39E-03	8.33E-05
6940086	GYPE	up	1.58	3.19E-01	6.94E-04
6100356	ALPL	up	1.55	1.47E-01	3.41E-04
380612	LOC644950	up	1.75	5.25E-03	8.33E-05
2070646	GPR84	up	1.51	9.15E-02	2.29E-04
130181	ANKRD22	up	1.70	6.70E-02	1.92E-04
5570398	FCGR1C	up	1.69	2.49E-02	1.11E-04
6620209	FCGR1B	up	1.79	1.80E-02	9.87E-05
5340161	TMEM217	up	1.52	7.18E-03	8.33E-05
1260348	MIR223	up	1.53	4.71E-02	1.53E-04
7100161	VNN2	up	1.63	3.63E-02	1.33E-04
110639	MAPK14	up	1.64	1.84E-02	9.87E-05
3180220	GPR109B	up	1.77	2.88E-03	8.33E-05
4860255	ASPRV1	up	1.57	7.99E-02	2.06E-04
1030463	PROK2	up	1.59	2.19E-02	1.05E-04
6560576	OCRL	up	1.53	5.62E-02	1.70E-04
3520327	LOC642161	down	-1.50	8.56E-02	2.17E-04
2760500	CD38	down	-1.72	2.51E-03	8.33E-05
7570196	TSPAN9	up	1.55	7.23E-02	2.01E-04
630167	SDCBP	up	1.53	1.81E-02	9.87E-05
2140707	SLPI	up	1.79	1.02E-01	2.49E-04
3850398	STX2	up	1.66	1.27E-03	8.33E-05
6290270	MNDA	up	1.54	8.06E-03	8.61E-05
4920612	GNLY	down	-2.06	1.44E-02	9.87E-05
6510333	LOC730286	up	1.55	3.64E-02	1.33E-04
1440209	RGS10	up	1.55	1.02E-01	2.50E-04
4220292	LOC646301	up	1.53	2.35E-02	1.09E-04
5090754	KIAA0101	down	-1.53	7.64E-02	2.02E-04
5130025	LOC100134703	up	1.53	1.44E-02	9.87E-05
3800021	LOC647506	down	-1.68	3.67E-02	1.33E-04
4880600	PTCRA	up	1.53	5.86E-02	1.73E-04
4670327	FBXO7	up	1.57	1.23E-01	2.89E-04
1740451	IL1R2	up	1.76	1.76E-01	4.01E-04
5560246	TPM1	up	1.66	8.32E-02	2.12E-04
4570255	LEF1	down	-1.53	6.89E-02	1.94E-04
3830228	GPR109A	up	1.79	1.49E-03	8.33E-05
5310445	KREMEN1	up	1.97	2.95E-02	1.18E-04
6550164	DEFA4	up	1.63	2.80E-01	6.19E-04
540703	RFESD	up	1.58	7.76E-02	2.03E-04
5080192	SERPINE2	up	1.57	3.68E-02	1.33E-04
460373	LACTB	up	1.51	1.89E-03	8.33E-05
1430711	KLF12	down	-1.51	2.93E-02	1.18E-04
1690706	HIST1H3H	up	1.58	5.81E-02	1.73E-04
380689	TSC22D1	up	1.52	4.52E-02	1.50E-04
6560398	NAT8B	up	1.73	2.81E-02	1.16E-04
1990037	CHI3L1	up	2.08	2.86E-02	1.17E-04
2030079	RUFY1	up	1.52	1.28E-02	9.78E-05
6900241	ABCB9	down	-1.85	7.52E-03	8.33E-05
5290598	ABCB9	down	-1.85	6.10E-03	8.33E-05
5080692	HLA-A29.1	up	2.09	1.77E-01	4.01E-04
3710553	SMOX	up	2.12	1.51E-02	9.87E-05
4640446	FHL1	up	1.53	7.82E-02	2.03E-04
7200044	SH3BGRL2	up	1.65	3.60E-02	1.33E-04
2000592	FTHL8	up	1.51	9.38E-02	2.34E-04
2230431	IRF5	up	1.63	5.64E-02	1.70E-04
4390398	LCN2	up	1.53	1.86E-01	4.18E-04
5360070	CCNB2	down	-1.51	6.21E-02	1.80E-04
3460053	SIPA1L2	up	1.51	7.39E-02	2.02E-04
3370594	LILRA2	up	1.54	1.85E-02	9.87E-05
7650497	ELANE	up	1.67	1.77E-01	4.01E-04
1260576	C1orf198	up	1.59	2.73E-02	1.13E-04
5310411	H2AFJ	up	1.64	1.59E-02	9.87E-05
5050402	HIST1H2BK	up	1.52	2.64E-02	1.11E-04
3440360	ZNF438	up	1.54	1.56E-02	9.87E-05
3780100	OSBP2	up	1.61	2.71E-01	6.01E-04
4060471	FAH	up	1.88	4.40E-03	8.33E-05
2750767	LOC100129960	up	1.57	1.92E-02	9.93E-05
540091	STEAP4	up	1.78	9.20E-03	8.78E-05
3420373	SOD2	up	1.74	8.55E-03	8.78E-05
3460376	RTN2	up	1.55	6.28E-03	8.33E-05
2190128	RAB32	up	1.59	2.26E-03	8.33E-05
940673	PACAP	down	-1.60	1.85E-02	9.87E-05
3830601	SLC2A11	up	1.55	3.07E-01	6.74E-04
150041	MANSC1	up	1.56	1.03E-01	2.51E-04
6580019	LOC731954	up	1.54	2.40E-02	1.09E-04
1050215	KCNJ15	up	1.93	6.40E-03	8.33E-05
270068	SMOX	up	1.90	2.09E-02	1.02E-04
1430484	C21orf81	down	-1.59	7.12E-02	1.99E-04
6960274	HAL	up	1.52	4.96E-02	1.56E-04
6280470	LOC728519	up	1.60	7.06E-02	1.98E-04
160070	C16orf30	down	-1.53	7.45E-02	2.02E-04
7550167	SV2A	up	1.57	4.12E-02	1.41E-04
7320424	HNRPA1L-2	down	-1.56	4.30E-02	1.45E-04
4670102	EGLN1	up	1.53	6.74E-03	8.33E-05
1050168	PI3	up	1.79	1.22E-01	2.89E-04
450753	HIST1H2BC	up	1.52	2.68E-02	1.12E-04
3390121	CASP5	up	1.59	8.12E-02	2.08E-04
6290747	NRGN	up	1.53	3.92E-02	1.36E-04
5890386	LOC642103	up	1.83	1.42E-02	9.87E-05
5420600	BEST1	up	1.56	1.99E-02	9.99E-05

2320689	LOC653610	up	1.81	1.06E-02	9.09E-05
4220110	LOC647450	down	-1.57	5.34E-02	1.65E-04
6290685	HIST1H2BJ	up	1.90	3.64E-04	8.33E-05
1770170	CTDSPL	up	1.51	1.09E-01	2.59E-04
4230678	HIST2H2BE	up	1.60	2.57E-02	1.11E-04
1340500	CLEC12A	up	1.55	7.47E-02	2.02E-04
3780358	CLEC12A	up	1.71	5.36E-02	1.65E-04
7150392	LOC100130623	down	-1.53	7.08E-03	8.33E-05
4860600	MAPK14	up	1.66	2.01E-02	9.99E-05
1770333	PDE5A	up	1.52	6.12E-02	1.79E-04
5270377	LOC728790	up	1.79	7.05E-03	8.33E-05
150609	LOC652493	down	-1.60	3.74E-02	1.33E-04
4010270	LOC440731	up	1.82	4.20E-03	8.33E-05
6620630	XK	up	1.90	1.76E-01	4.01E-04
4560021	LOC100132112	up	1.54	3.38E-02	1.30E-04
2710709	FCGR1B	up	1.82	1.57E-02	9.87E-05
4180079	CRISPLD2	up	1.55	3.69E-02	1.33E-04
2490142	C19orf35	up	1.51	7.38E-02	2.02E-04
7510253	ACRBP	up	1.54	5.65E-02	1.70E-04
4640092	IMPDH1	up	1.60	4.75E-04	8.33E-05
3450537	DGAT2	up	1.63	1.84E-02	9.87E-05
4670575	HTATIP2	up	1.52	2.52E-03	8.33E-05
3060523	NAMPT	up	1.52	4.65E-02	1.52E-04
2230379	NAMPT	up	1.56	3.66E-02	1.33E-04
1260482	GZMK	down	-1.69	5.32E-03	8.33E-05
2810280	SLC6A6	up	1.69	6.40E-03	8.33E-05
2450647	KRT1	up	2.09	1.00E-01	2.46E-04
1850546	FAM102A	down	-1.67	1.22E-02	9.50E-05
3440630	ESPN	up	1.71	7.75E-02	2.03E-04
7160474	HLA-DQB1	up	1.89	6.53E-02	1.88E-04
5390497	C7orf53	up	1.67	5.60E-03	8.33E-05
4640427	HIATL1	up	1.54	2.31E-03	8.33E-05
520523	CYP4F3	up	1.68	3.80E-02	1.34E-04
1070367	C19orf59	up	2.39	7.52E-03	8.33E-05
1050292	GP9	up	1.54	7.60E-02	2.02E-04
6480630	ATP9A	up	1.72	1.76E-02	9.87E-05
6330612	LOC100133875	up	1.55	3.53E-02	1.33E-04
70343	FRAT1	up	1.60	1.79E-02	9.87E-05
1500010	CDC20	down	-1.81	6.93E-03	8.33E-05
5560193	BCAS4	down	-1.51	2.37E-02	1.09E-04
4290079	BCAS4	down	-1.63	1.29E-02	9.78E-05
2480192	SESN3	up	1.63	1.78E-01	4.02E-04
3390612	TLR8	up	1.54	1.66E-02	9.87E-05
4880370	JUP	down	-1.87	6.26E-02	1.81E-04
3130541	CCNF	down	-1.53	1.17E-02	9.39E-05
150626	LOC641710	up	1.66	2.77E-03	8.33E-05
610154	CLDND2	down	-1.58	3.70E-02	1.33E-04
2360136	LOC440926	up	1.55	8.86E-03	8.78E-05
2360095	TNFRSF10B	up	1.54	1.67E-02	9.87E-05
4210280	PGCP	up	1.51	1.40E-02	9.87E-05
5260754	TSC22D1	up	1.68	3.44E-02	1.31E-04
1710553	HSPA6	up	1.51	1.79E-02	9.87E-05
2680189	LAG3	down	-1.78	4.83E-03	8.33E-05
50136	CMTM5	up	1.57	7.56E-02	2.02E-04
1230164		up	1.56	5.60E-03	8.33E-05
5050138	NT5M	up	1.61	5.74E-02	1.72E-04
5810612	ANPEP	up	1.60	4.81E-02	1.54E-04
3710025	RUFY1	up	1.55	8.90E-03	8.78E-05
5310437	MYL9	up	1.76	3.91E-02	1.36E-04
5340468	ITGA2B	up	1.58	1.49E-01	3.43E-04
3390048	LIMK2	up	1.68	1.09E-02	9.09E-05
5910632	SMARCD3	up	1.76	2.42E-03	8.33E-05
5260349	NGFRAP1	up	1.66	2.44E-02	1.09E-04
6860220	NGFRAP1	up	1.58	3.48E-02	1.32E-04
2230563	PPBP	up	1.55	7.73E-02	2.03E-04
1430524	LTA4H	up	1.52	1.90E-02	9.89E-05
1240358	RAB20	up	1.58	1.09E-02	9.09E-05
7040735	CYP27A1	up	1.72	4.40E-02	1.47E-04
4290243	ARHGAP24	up	1.52	1.61E-02	9.87E-05
4290148	HIST2H2AA4	up	1.79	1.10E-02	9.09E-05
3170601	CLEC12A	up	1.59	6.88E-02	1.94E-04
3130220	TMEM158	up	1.73	1.27E-01	2.97E-04
450609	IGLL3	down	-1.84	9.30E-03	8.78E-05
3780187	GYPB	up	1.67	3.07E-01	6.74E-04
4180544	ROPN1L	up	1.64	1.44E-02	9.87E-05
3460204	LOC730235	up	1.56	1.54E-02	9.87E-05
3400672	SERPINB8	up	1.61	2.68E-03	8.33E-05
4260338	LOC647307	down	-1.52	2.52E-03	8.33E-05
2070369	TREML1	up	1.69	4.57E-02	1.50E-04
3370327	MOSC1	up	1.78	6.24E-03	8.33E-05
1980431	DOK3	up	1.56	6.27E-03	8.33E-05
620091	LOC730234	up	1.78	6.81E-04	8.33E-05
1820592	HIST2H2AA3	up	1.76	1.20E-02	9.46E-05
610451	HIST2H2AA3	up	1.92	6.19E-03	8.33E-05
1940504	FKBP1A	up	1.53	9.33E-04	8.33E-05
7000546	LILRA6	up	1.61	5.26E-02	1.64E-04
3060092	LAT2	up	1.65	2.86E-03	8.33E-05
450348	GNG10	up	1.50	2.53E-02	1.11E-04
4570725	LOC653604	up	1.64	3.03E-03	8.33E-05
6420164	ST6GALNAC2	up	1.56	2.40E-02	1.09E-04
1470132	B3GNT8	up	1.51	3.35E-02	1.30E-04
1300431	LY6G6F	up	1.51	9.51E-02	2.36E-04
2000451	PYHIN1	down	-1.56	4.42E-02	1.47E-04
4200746	BPI	up	1.64	1.55E-01	3.56E-04
4200754	ST3GAL4	up	1.60	1.61E-02	9.87E-05
5050274	DHRS12	up	1.63	1.10E-02	9.09E-05

3710086	DHRS12	up	1.57	4.13E-02	1.41E-04
2230241	F13A1	up	1.53	5.41E-02	1.65E-04
620544	HLA-DRB6	down	-1.87	6.84E-02	1.94E-04
4010564	TRPM6	up	1.62	2.96E-02	1.18E-04
940274	GK	up	1.58	1.90E-02	9.89E-05
6520594	ARHGEF11	up	1.56	4.98E-03	8.33E-05
7150070	ARHGEF11	up	1.52	1.10E-02	9.09E-05
6380672	CA4	up	1.58	1.06E-01	2.56E-04
4290110	CYP4F3	up	1.61	2.18E-02	1.05E-04
1070450	PTGS1	up	1.53	7.63E-02	2.02E-04
6590437	FCGR2A	up	1.76	1.48E-03	8.33E-05
240441	IL1R2	up	1.63	1.25E-01	2.92E-04
2450110	MMP23B	down	-1.56	1.82E-02	9.87E-05
6900091	IKZF3	down	-1.66	2.53E-02	1.11E-04
5390246	CCR7	down	-1.53	1.40E-01	3.25E-04
6660162	LRG1	up	1.54	3.00E-02	1.18E-04
1450484	HIST1H2BE	up	1.51	4.28E-02	1.45E-04
990349	MCTP2	up	1.60	1.54E-02	9.87E-05
2340577	AQP10	up	1.83	7.79E-02	2.03E-04
3180609	LOC100008588	down	-1.62	3.97E-01	8.58E-04
1240450	CD27	down	-1.71	5.20E-03	8.33E-05
6100022	HIST2H2AC	up	1.70	1.30E-02	9.78E-05
6280170	PDCD1	down	-1.53	5.97E-02	1.76E-04
3060682	BPGM	up	1.54	3.78E-01	8.19E-04
4920523	HOXC6	up	1.57	1.05E-02	9.09E-05
5260424	GPR97	up	1.55	5.37E-02	1.65E-04
3940438	NCF1	up	1.53	7.47E-03	8.33E-05
5870136	CLIC3	down	-1.51	5.07E-02	1.59E-04
6400674	LOC730387	up	1.53	3.94E-02	1.36E-04
2650114	ITGB5	up	1.51	7.49E-02	2.02E-04
520086	FCGR1A	up	1.71	3.24E-02	1.27E-04
6840674	ZNF185	up	1.60	2.02E-02	9.99E-05
630619	HPSE	up	1.61	2.24E-02	1.06E-04
6290672	LOC728417	up	1.61	6.47E-03	8.33E-05
430546	HIST1H2BG	up	1.76	1.58E-02	9.87E-05
5570039	LOC728744	up	1.93	2.43E-02	1.09E-04
4860220	SRGAP2	down	-1.65	9.37E-03	8.78E-05
3180528	MMP9	up	1.88	4.30E-02	1.45E-04
6130075	LOC391769	up	1.62	5.81E-03	8.33E-05
7320270	PNMA3	down	-1.76	4.83E-03	8.33E-05
5560414	LOC440093	up	1.53	1.09E-02	9.09E-05
460463	SMARCD3	up	1.94	2.88E-03	8.33E-05
1260133	LOC642334	up	1.51	2.04E-02	1.00E-04
6480142	LAMP2	up	1.55	1.86E-02	9.87E-05
5870678	LOC441763	down	-1.56	3.19E-01	6.94E-04
7320678		up	1.58	9.45E-02	2.35E-04
3850112	RBP7	up	1.65	4.47E-03	8.33E-05
2490333	ZNF467	up	1.50	7.34E-03	8.33E-05
7100703	LOC100132287	up	1.64	1.50E-02	9.87E-05
2810162	LAT2	up	1.72	1.47E-03	8.33E-05
4210113	CMTM2	up	1.58	7.28E-02	2.01E-04
5860551	BTNL8	up	1.88	7.84E-03	8.53E-05
270240	SLC26A8	up	1.57	9.93E-02	2.45E-04
4850398	TXNDC5	down	-1.77	3.76E-02	1.33E-04
2060411	LOC652616	up	1.64	3.51E-03	8.33E-05
4220187	DYSF	up	1.58	1.94E-02	9.93E-05
6200370	LILRA3	up	1.62	2.31E-02	1.08E-04
3930008	RPL14	down	-1.81	6.22E-02	1.80E-04
4120367	LPCAT2	up	1.59	4.85E-02	1.54E-04
610519	TPM1	up	1.62	1.09E-01	2.59E-04
3520601	MPO	up	1.76	8.72E-02	2.20E-04
580136	LOC652694	down	-1.67	2.99E-02	1.18E-04
1430435	C3orf34	up	1.51	8.29E-03	8.70E-05
1410221	S100A12	up	1.78	8.05E-02	2.07E-04
3940685	SIGLEC9	up	1.52	6.86E-03	8.33E-05
1740360	USP18	down	-1.68	8.97E-02	2.26E-04
6040259	ALOX12	up	2.00	6.88E-03	8.33E-05
770682	MGC29506	down	-1.71	2.64E-02	1.11E-04
4150369	GK	up	1.56	1.83E-02	9.87E-05
4900309	MGC13057	up	1.67	1.23E-01	2.89E-04
2260725	MGAM	up	1.70	2.61E-02	1.11E-04
4670458	Sep-04	up	1.50	2.68E-01	5.97E-04
240594	ANXA3	up	2.17	2.27E-02	1.07E-04
5270338	ALAS2	up	1.53	2.98E-01	6.57E-04
4610538	SUMO1P1	up	1.58	4.67E-02	1.52E-04
2120682	GPR89C	down	-1.50	1.54E-02	9.87E-05
6400603	PVRIG	down	-1.65	9.29E-03	8.78E-05
6130441	ASPM	down	-1.58	2.90E-02	1.18E-04
7560593	OSM	up	1.64	1.03E-02	9.09E-05
4540241	C5orf32	up	1.60	1.05E-01	2.54E-04
2260189	FLJ14166	up	1.60	2.59E-02	1.11E-04
2340241	IMPA2	up	1.60	1.96E-02	9.96E-05
7610767	F5	up	1.58	4.82E-02	1.54E-04
840253	ALDH2	up	1.70	1.68E-02	9.87E-05
520609	KIAA1324	up	2.22	1.66E-02	9.87E-05
2100528	HIST1H3F	up	1.54	4.79E-02	1.54E-04
2970019	HIST1H4H	up	1.60	7.38E-02	2.02E-04
4250753	TSPAN33	up	1.53	1.86E-02	9.87E-05
2190139	CA1	up	1.79	2.22E-01	4.97E-04
2680605	IGJ	down	-2.05	1.15E-02	9.30E-05
610437	CD24	up	1.55	1.22E-01	2.89E-04
