# Supplementary material for: Inflammasome Activation Underlying Central Nervous System Deterioration in HIV-Associated Tuberculosis
Source: J Infect Dis. 2016 Dec 8;215(5):677–86. doi: 10.1093/infdis/jiw561 (PMC5388298; doi:10.1093/infdis/jiw561)
Supplement: SupplementaryTableS3 [file jiw561_suppl_SupplementaryTableS3.pdf]

Illumina_ProbeID	Symbol	Regulation	FC	p-value	q-value
2190349	KCNJ15	up	2.57	1.64E-03	1.56E-04
5570139	QPCT	up	1.53	4.76E-02	3.19E-04
5960653	TMEM55A	up	1.51	3.23E-02	2.48E-04
5900050	LOC653800	down	-1.69	1.81E-02	1.73E-04
380612	LOC644950	up	1.53	4.32E-02	2.99E-04
2070646	GPR84	up	1.61	7.75E-02	4.58E-04
130181	ANKRD22	up	2.93	1.29E-03	1.56E-04
430605	TMEM137	down	-1.56	7.01E-03	1.56E-04
4280026	ACAP2	up	1.51	2.12E-02	1.93E-04
4290368	PSTPIP2	up	1.57	3.29E-02	2.49E-04
70167	LY96	up	1.59	2.63E-02	2.22E-04
2630195	VAMP5	up	1.63	6.51E-03	1.56E-04
5570398	FCGR1C	up	1.94	7.78E-03	1.56E-04
6620209	FCGR1B	up	2.15	1.17E-02	1.62E-04
130609	FCGBP	down	-1.54	1.04E-01	5.71E-04
1510364	GBP5	up	1.79	8.00E-03	1.56E-04
6270681	BST1	up	1.58	2.19E-02	1.96E-04
1260348	MIR223	up	1.51	5.34E-02	3.41E-04
1850220	DAPP1	up	1.77	1.11E-03	1.56E-04
6560020	COMMD2	up	1.62	5.11E-03	1.56E-04
2490315	NA	up	1.54	1.21E-01	6.37E-04
7100161	VNN2	up	1.60	4.42E-02	3.01E-04
110639	MAPK14	up	1.74	8.46E-03	1.56E-04
3450349	CSF2RB	up	1.58	8.72E-03	1.56E-04
3180220	GPR109B	up	1.92	3.02E-03	1.56E-04
4860255	ASPRV1	up	1.82	1.79E-02	1.73E-04
2190452	PIM3	up	1.53	4.42E-03	1.56E-04
5860064	FCHO2	up	1.56	4.83E-02	3.20E-04
2940446	TGM2	up	1.69	1.31E-01	6.77E-04
2030309	SERPING1	up	1.80	9.08E-02	5.13E-04
6770707	SQRDL	up	1.50	1.51E-02	1.62E-04
3170273	FER1L3	up	1.95	1.42E-02	1.62E-04
7320377	LOC284422	up	1.65	1.95E-01	9.64E-04
3780017	SIRPB1	up	1.70	1.99E-03	1.56E-04
4780044	LOC389386	up	1.79	3.05E-02	2.42E-04
670470	SLK	up	1.69	1.84E-03	1.56E-04
4060021	SLK	up	1.62	6.46E-03	1.56E-04
4920612	GNLY	down	-1.89	2.82E-02	2.30E-04
1450504	NA	down	-1.64	9.61E-03	1.59E-04
4810468	SELP	up	1.50	1.03E-01	5.70E-04
2190475	DNM3	up	1.64	2.09E-02	1.92E-04
3610743	SF1	down	-1.66	8.75E-03	1.56E-04
7650451	ZNF683	down	-1.65	1.34E-01	6.86E-04
4120561	LIMK2	up	1.53	2.50E-02	2.16E-04
4810112	HAUS4	up	1.57	8.96E-03	1.57E-04
1450608	VWCE	up	1.84	6.31E-02	3.92E-04
940220	NOD2	up	1.70	2.53E-03	1.56E-04
4670327	FBXO7	up	1.50	1.90E-01	9.45E-04
2940767	CEBPE	down	-1.55	8.73E-02	4.97E-04
4670592	MYOF	up	2.22	3.91E-03	1.56E-04
6290475	HECW2	up	1.51	1.32E-01	6.81E-04
4780600	RBM47	up	1.54	1.37E-02	1.62E-04
7650195	LOC727908	down	-1.57	1.06E-02	1.61E-04
3060520	LOC642073	up	1.59	1.06E-01	5.76E-04
5560246	TPM1	up	1.56	5.54E-02	3.51E-04
4590224	EVI2A	up	1.54	1.12E-02	1.62E-04
2680162	EVI2A	up	1.51	3.42E-02	2.54E-04
3830228	GPR109A	up	1.84	3.68E-03	1.56E-04
3400392	FPR2	up	1.81	1.58E-02	1.62E-04
3390301	KREMEN1	up	1.73	8.05E-02	4.65E-04
5310754	VNN1	up	1.71	1.08E-01	5.81E-04
6840035	GBP1	up	1.80	1.01E-02	1.61E-04
2190148	GBP1	up	1.77	6.40E-03	1.56E-04
240053	GCH1	up	2.06	2.32E-03	1.56E-04
3800398	FBXO6	up	1.54	1.23E-02	1.62E-04
460373	LACTB	up	1.57	2.75E-03	1.56E-04
770494	TSC22D3	up	1.51	1.06E-01	5.76E-04
4860280	NA	down	-1.53	3.90E-02	2.77E-04
240750	TELO2	up	1.54	1.17E-02	1.62E-04
4730059	BATF2	up	1.96	1.53E-02	1.62E-04
1990037	CHI3L1	up	1.61	1.68E-01	8.44E-04
5340427	TLR6	up	1.50	2.83E-02	2.30E-04
840068	C3AR1	up	1.62	3.87E-02	2.76E-04
7050519	CLEC4D	up	1.59	9.31E-02	5.22E-04
6510735	DDX60L	up	1.64	1.50E-02	1.62E-04
7510367	GPR160	up	1.54	8.30E-02	4.78E-04
4640446	FHL1	up	1.61	2.38E-02	2.09E-04
730528	PLAUR	up	1.58	3.61E-02	2.66E-04
830750	NCF1B	up	1.56	9.41E-03	1.59E-04
2230431	IRF5	up	1.53	1.10E-01	5.88E-04
4390398	LCN2	up	1.53	2.50E-01	1.21E-03
3460053	SIPA1L2	up	1.64	4.69E-02	3.16E-04
1240270	LMTK2	up	1.61	1.75E-02	1.71E-04
1580435	TGM2	up	1.51	1.69E-01	8.46E-04
1260576	C1orf198	up	1.52	2.15E-02	1.95E-04
4780128	ATF3	up	1.86	2.90E-02	2.33E-04
2320576	RBM47	up	1.51	1.14E-02	1.62E-04
160019	SORT1	up	1.61	7.74E-03	1.56E-04
5050402	HIST1H2BK	up	1.57	6.63E-02	4.07E-04
5900468	HDAC4	up	1.58	6.42E-03	1.56E-04
2850315	ORM1	up	1.68	1.34E-01	6.86E-04
6100136	CASP4	up	1.56	3.68E-02	2.70E-04
6420750	TLR2	up	1.63	6.67E-02	4.08E-04
540347	ITM2B	up	1.52	2.65E-02	2.22E-04
2750767	LOC100129960	up	1.54	2.75E-02	2.29E-04
5270753	ARG1	up	1.55	2.67E-01	1.28E-03
6520451	TLR1	up	1.58	4.89E-03	1.56E-04
540091	STEAP4	up	1.68	1.46E-02	1.62E-04
2750685	LOC392437	up	1.54	1.09E-02	1.61E-04
3420373	SOD2	up	1.61	1.56E-02	1.62E-04
6250497	FBXL5	up	1.58	5.51E-03	1.56E-04
730221	PTPLA	up	1.59	1.31E-01	6.77E-04
1410278	PF4V1	up	1.57	7.14E-02	4.29E-04
150041	MANSC1	up	1.53	1.10E-01	5.88E-04
1050215	KCNJ15	up	2.08	3.49E-03	1.56E-04
4570164	LOC389386	up	1.50	6.33E-02	3.92E-04
3850202	IGSF6	up	1.51	1.23E-02	1.62E-04
620403	LOC400759	up	1.66	3.42E-02	2.54E-04
6280470	LOC728519	up	1.60	7.40E-02	4.44E-04
1070593	KLHL2	up	1.55	4.02E-02	2.84E-04
4610184	NA	down	-1.51	4.34E-02	2.99E-04

4760747	TPST1	up	1.82	1.01E-01	5.63E-04
3800592	LOC100129697	up	1.68	1.31E-03	1.56E-04
520451	NA	down	-1.51	3.06E-02	2.42E-04
6370228	NA	down	-1.62	6.86E-02	4.17E-04
650743	C10orf11	up	1.59	3.27E-03	1.56E-04
6110634	ERLIN1	up	1.86	1.59E-02	1.62E-04
5310224	LOC100133678	up	1.74	1.04E-01	5.71E-04
3390121	CASP5	up	1.78	3.28E-02	2.49E-04
5890386	LOC642103	up	1.77	1.26E-02	1.62E-04
5960239	CCR2	up	1.51	5.38E-03	1.56E-04
3520438	ARAP2	up	1.52	1.70E-03	1.56E-04
2140288	FLJ22662	up	1.53	7.37E-03	1.56E-04
2320689	LOC653610	up	1.62	4.24E-02	2.95E-04
6290685	HIST1H2BJ	up	1.56	3.31E-02	2.49E-04
3780358	CLEC12A	up	1.53	1.13E-01	6.02E-04
6180543	GK5	down	-1.61	1.49E-02	1.62E-04
1230309	RPL26	up	1.57	8.34E-02	4.78E-04
4860600	MAPK14	up	1.82	1.10E-02	1.61E-04
5900594	CD58	up	1.66	5.63E-03	1.56E-04
3610035	CD58	up	1.59	1.45E-02	1.62E-04
4040519	LOC728059	up	1.51	2.27E-02	2.01E-04
5270377	LOC728790	up	1.60	4.14E-02	2.91E-04
4010270	LOC440731	up	1.51	4.94E-02	3.23E-04
460672	TMEM165	up	1.70	8.83E-04	1.56E-04
60470	STX11	up	1.76	7.16E-03	1.56E-04
1010592	CD36	up	1.67	3.21E-03	1.56E-04
2710709	FCGR1B	up	2.21	6.78E-03	1.56E-04
5490601	SLC40A1	up	1.63	9.04E-03	1.57E-04
4180079	CRISPLD2	up	1.59	1.39E-02	1.62E-04
3140520	TMOD1	up	1.66	2.10E-01	1.03E-03
7510253	ACRBP	up	1.60	1.52E-02	1.62E-04
2230379	NAMPT	up	1.62	1.25E-02	1.62E-04
2450647	KRT1	up	1.91	1.16E-01	6.14E-04
360132	LHFPL2	up	1.81	1.09E-02	1.61E-04
3780066	LOC646438	up	1.53	2.10E-02	1.92E-04
7160474	HLA-DQB1	up	2.47	7.92E-03	1.56E-04
4640427	HIATL1	up	1.71	6.58E-04	1.56E-04
3390689	SMCHD1	up	1.55	1.64E-02	1.64E-04
1440615	OTOF	down	-1.76	2.36E-01	1.15E-03
4640561	NA	down	-1.55	4.92E-02	3.23E-04
1070367	C19orf59	up	1.54	2.22E-01	1.09E-03
7100717	RPS15A	up	1.54	6.88E-02	4.17E-04
6330612	LOC100133875	up	1.51	6.95E-02	4.20E-04
2640670	IDI1	up	1.50	8.79E-03	1.56E-04
1030431	ACSL1	up	1.63	1.53E-02	1.62E-04
3360132	SNX20	up	1.53	1.55E-02	1.62E-04
1050068	F2RL1	up	1.60	1.14E-02	1.62E-04
5810047	F2RL1	up	1.65	1.36E-02	1.62E-04
5560075	MFGE8	down	-1.61	1.31E-02	1.62E-04
5560561	TNS3	up	1.56	4.97E-03	1.56E-04
7570600	FLJ33590	down	-1.67	1.41E-02	1.62E-04
60719	KCNJ2	up	1.54	1.06E-02	1.61E-04
7000307	ACSL4	up	1.63	5.97E-03	1.56E-04
5340767	CEACAM1	up	1.55	1.06E-01	5.76E-04
1230307	RRBP1	down	-1.79	6.03E-03	1.56E-04
110372	CSTA	up	1.60	1.23E-02	1.62E-04
1500010	CDC20	down	-1.56	8.03E-02	4.65E-04
2480192	SESN3	up	1.74	1.10E-01	5.88E-04
3390612	TLR8	up	1.66	9.45E-03	1.59E-04
3140064	C22orf34	down	-1.71	1.52E-02	1.62E-04
4060452	LOC648226	down	-2.21	2.67E-03	1.56E-04
3290730	LOC100133591	up	1.78	1.06E-01	5.76E-04
2640377	PGCP	up	1.57	4.63E-03	1.56E-04
4210280	PGCP	up	1.51	2.77E-02	2.29E-04
1400593	SIGLEC14	down	-3.10	4.91E-02	3.23E-04
3830497	KLF12	down	-1.65	1.59E-02	1.62E-04
4150543	FPR2	up	1.72	8.48E-03	1.56E-04
2030678	HIST2H2AB	up	1.90	2.90E-02	2.33E-04
3780594	LOC440345	down	-1.63	2.86E-03	1.56E-04
50136	CMTM5	up	1.53	9.25E-02	5.20E-04
3420026	FAS	up	1.86	8.76E-04	1.56E-04
5310437	MYL9	up	1.71	2.23E-02	1.99E-04
2480327	MYL9	up	1.95	1.73E-02	1.70E-04
7040142	KYNU	up	1.61	4.29E-03	1.56E-04
5910632	SMARCD3	up	1.82	1.63E-03	1.56E-04
5260349	NGFRAP1	up	1.52	1.99E-02	1.89E-04
6860220	NGFRAP1	up	1.52	1.68E-02	1.67E-04
2230563	PPBP	up	1.67	3.80E-02	2.72E-04
2450070	LOC440345	down	-1.53	5.08E-02	3.30E-04
4900747	C11orf75	up	1.61	5.04E-03	1.56E-04
6400332	CNIH4	up	1.53	1.56E-02	1.62E-04
1240358	RAB20	up	1.74	1.63E-02	1.64E-04
1990300	SOCS1	up	1.93	1.00E-02	1.61E-04
6250484	CFH	up	1.80	4.71E-02	3.17E-04
4290148	HIST2H2AA4	up	1.66	2.46E-02	2.13E-04
3130220	TMEM158	up	1.68	6.01E-02	3.78E-04
3290292	LAP3	up	1.59	3.26E-02	2.49E-04
1500735	CTSG	down	-1.62	2.95E-01	1.41E-03
4850592	P2RY14	up	2.25	8.57E-03	1.56E-04
6510170	IFIT3	up	1.57	1.54E-01	7.79E-04
3460204	LOC730235	up	1.52	3.08E-02	2.43E-04
3400672	SERPINB8	up	1.56	5.73E-03	1.56E-04
5570711	IDO1	up	1.80	1.61E-01	8.10E-04
3370327	MOSC1	up	1.51	5.81E-02	3.66E-04
4150270	ANKRD22	up	2.47	2.20E-03	1.56E-04
6420008	PROS1	up	1.93	1.27E-02	1.62E-04
2140524	HIST1H3D	up	1.56	9.08E-02	5.13E-04
3060358	NA	down	-1.72	8.35E-03	1.56E-04
3130543	RNASE3	down	-1.66	1.52E-01	7.71E-04
4780068	SULT1B1	up	1.51	1.13E-01	6.02E-04
610451	HIST2H2AA3	up	1.80	2.04E-02	1.89E-04
3310538	CD36	up	1.53	2.03E-02	1.89E-04
6370717	GNG10	up	1.71	7.47E-03	1.56E-04
450348	GNG10	up	1.73	1.57E-02	1.62E-04
4570725	LOC653604	up	1.61	1.37E-02	1.62E-04
2000286	C8orf16	down	-1.95	4.86E-03	1.56E-04
5050274	DHRS12	up	1.63	2.19E-02	1.96E-04
6590646	FAM26F	up	1.80	1.17E-02	1.62E-04
1070477	ALDH1A1	up	1.69	7.26E-03	1.56E-04
3780544	SF3B14	up	1.55	1.45E-02	1.62E-04
830615	ZDHHC19	up	1.62	2.51E-01	1.21E-03
2230241	F13A1	up	1.63	3.12E-02	2.43E-04

650563	FAHD1	up	1.52	6.10E-02	3.82E-04
3460685	KYNU	up	1.54	1.23E-02	1.62E-04
940274	GK	up	1.71	5.63E-03	1.56E-04
3940189	JAK2	up	1.59	1.44E-02	1.62E-04
1980523	PSCDBP	up	1.79	2.28E-04	1.56E-04
6590437	FCGR2A	up	1.52	2.01E-02	1.89E-04
2900139	FLJ20489	up	1.52	8.60E-02	4.91E-04
3450092	PELI1	up	1.51	7.76E-02	4.58E-04
6900091	IKZF3	down	-1.64	1.34E-02	1.62E-04
540392	REPS2	up	1.51	1.02E-01	5.66E-04
1450484	HIST1H2BE	up	1.56	4.44E-02	3.01E-04
2340577	AQP10	up	1.80	4.99E-02	3.26E-04
3180609	LOC100008588	down	-1.75	2.46E-01	1.20E-03
1240450	CD27	down	-1.68	8.32E-03	1.56E-04
6100022	HIST2H2AC	up	1.58	3.20E-02	2.47E-04
7510019	FOXP1	down	-1.52	3.69E-02	2.70E-04
4610129	RETN	up	1.66	1.87E-01	9.30E-04
2510551	STK3	up	1.61	1.42E-02	1.62E-04
1660113	FNDC3A	up	1.55	3.41E-03	1.56E-04
1230731	NFXL1	up	1.53	1.43E-01	7.33E-04
7400152	P2RY10	down	-1.51	3.11E-02	2.43E-04
3060747	CENTB2	up	1.53	1.03E-02	1.61E-04
360689	HEBP1	up	1.52	4.81E-03	1.56E-04
3140487	BMX	up	2.29	8.14E-03	1.56E-04
6760445	LAT1-3TM	down	-1.51	8.77E-03	1.56E-04
2490687	JMJD1C	up	1.51	4.87E-03	1.56E-04
5360243	ITGB5	up	1.58	3.18E-02	2.47E-04
520086	FCGR1A	up	1.92	1.03E-02	1.61E-04
4560047	CD74	up	1.67	1.71E-02	1.69E-04
6770131	OLFM4	up	1.71	3.20E-01	1.52E-03
5560471	FCAR	up	1.76	5.37E-02	3.42E-04
2810059	KLF4	up	1.59	1.33E-03	1.56E-04
6860164	CLEC1B	up	1.56	1.27E-01	6.63E-04
430546	HIST1H2BG	up	1.50	6.36E-02	3.92E-04
6480274	LOC730995	down	-1.54	4.42E-02	3.01E-04
1340128	HSDL2	up	1.56	1.76E-03	1.56E-04
1230068	PPP1R3D	up	1.54	1.35E-02	1.62E-04
5290068	SLFN13	down	-1.57	7.97E-03	1.56E-04
5570039	LOC728744	up	2.30	2.92E-03	1.56E-04
5870138	VWF	up	1.63	2.41E-02	2.11E-04
3710068	WARS	up	1.70	8.47E-03	1.56E-04
3180528	MMP9	up	1.67	7.95E-02	4.63E-04
7320270	PNMA3	down	-1.60	5.60E-03	1.56E-04
460463	SMARCD3	up	2.07	5.05E-04	1.56E-04
290301	LOC653352	down	-1.70	7.96E-03	1.56E-04
2000564	C6orf204	down	-1.51	9.91E-03	1.61E-04
5390487	SPTBN1	down	-1.78	2.56E-02	2.18E-04
6370458	TCP11L2	up	1.52	1.49E-01	7.58E-04
6220739	GRAMD1B	up	1.62	7.45E-02	4.45E-04
6510754	ALDH1A1	up	1.94	1.28E-03	1.56E-04
4060156	LOC100130492	down	-1.56	2.43E-02	2.12E-04
4210113	CMTM2	up	1.52	3.80E-02	2.72E-04
4860746	LOC642684	up	1.55	5.27E-02	3.41E-04
5960747	TRIM22	up	1.56	4.81E-02	3.20E-04
2060411	LOC652616	up	1.53	3.43E-02	2.54E-04
4860224	WARS	up	1.58	1.15E-02	1.62E-04
1770243	IGF2BP3	up	1.81	1.46E-03	1.56E-04
6450504	FLJ43093	down	-1.60	5.34E-02	3.41E-04
6200370	LILRA3	up	1.53	7.91E-02	4.63E-04
520408	IFIT3	up	1.58	1.27E-01	6.63E-04
4120367	LPCAT2	up	1.79	2.64E-02	2.22E-04
4260253	OBFC2A	up	1.51	9.58E-03	1.59E-04
610519	TPM1	up	1.51	7.86E-02	4.62E-04
1410221	S100A12	up	1.91	3.74E-02	2.71E-04
3460161	RAB3IL1	up	1.80	9.43E-02	5.27E-04
6270553	CXCL10	up	1.93	4.36E-02	2.99E-04
4150369	GK	up	1.69	5.59E-03	1.56E-04
2230204	OAS2	up	1.56	2.78E-02	2.29E-04
5270148	PLEK2	up	1.94	6.22E-02	3.88E-04
6350634	DYNLT1	up	1.64	2.56E-03	1.56E-04
3360634	SNX10	up	1.51	6.49E-03	1.56E-04
2260725	MGAM	up	1.88	7.35E-03	1.56E-04
4670458	Sep-04	up	2.05	3.72E-02	2.71E-04
240594	ANXA3	up	2.15	1.42E-02	1.62E-04
1260136	MEGF9	up	1.50	2.83E-02	2.30E-04
780403	HLA-DQA1	up	1.55	2.19E-01	1.08E-03
4780075	CEACAM8	up	1.61	3.11E-01	1.49E-03
4920328	SH3KBP1	down	-1.69	2.55E-02	2.18E-04
7380626	FLJ20273	up	1.59	1.64E-02	1.64E-04
3610343	NBN	up	1.55	1.28E-02	1.62E-04
510347	C9orf72	up	1.58	7.57E-03	1.56E-04
7570022	C9orf72	up	1.61	4.88E-03	1.56E-04
4730634	SLC31A2	up	1.59	1.09E-02	1.61E-04
3850441	MAZ	up	1.59	3.03E-02	2.42E-04
7560593	OSM	up	1.70	7.23E-03	1.56E-04
4540241	C5orf32	up	1.62	4.22E-02	2.95E-04
3890193	CLIC4	up	1.65	6.52E-03	1.56E-04
520609	KIAA1324	up	2.06	1.03E-02	1.61E-04
6220671	PLAUR	up	1.59	1.78E-02	1.72E-04
360475	PLAUR	up	1.51	1.29E-02	1.62E-04
2970019	HIST1H4H	up	1.55	1.26E-01	6.62E-04
870563	RNF138	up	1.81	1.11E-04	1.56E-04
1110102	HS2ST1	up	1.51	1.79E-03	1.56E-04
3780047	GBP6	up	1.99	5.33E-02	3.41E-04
3450736	LOC100133583	up	1.53	7.97E-02	4.63E-04
2190139	CA1	up	2.02	1.61E-01	8.11E-04
6060142	AFF1	up	1.57	1.53E-02	1.62E-04
2120451	CD46	up	1.55	1.20E-02	1.62E-04
2650605	C4orf18	up	1.59	3.80E-02	2.72E-04
2340500	KLC3	up	1.55	2.03E-01	1.00E-03
5050768	LONRF1	up	1.62	2.23E-03	1.56E-04
7160468	DHRS9	up	1.62	7.63E-02	4.54E-04
4070424	C16orf93	down	-1.57	5.53E-03	1.56E-04
2140242	TNFAIP6	up	1.61	2.01E-02	1.89E-04
