# Supplementary material for: Inflammasome Activation Underlying Central Nervous System Deterioration in HIV-Associated Tuberculosis
Source: J Infect Dis. 2016 Dec 8;215(5):677–86. doi: 10.1093/infdis/jiw561 (PMC5388298; doi:10.1093/infdis/jiw561)
Supplement: SupplementaryTableS4 [file jiw561_suppl_SupplementaryTableS4.pdf]

Illumina_ProbeID	Symbol	Regulation	FC	p-value	q-value
6840035	GBP1	up	1.80	1.01E-02	1.78E-05
1510364	GBP5	up	1.79	8.00E-03	1.78E-05
940220	NOD2	up	1.70	2.53E-03	1.78E-05
3390612	TLR8	up	1.66	9.45E-03	1.78E-05
6520451	TLR1	up	1.58	4.89E-03	1.78E-05
840685	IL1B	up	1.47	1.64E-02	1.78E-05
2490161	CLEC2B	up	1.43	1.77E-02	1.78E-05
450491	CASP1	up	1.38	2.20E-02	1.78E-05
5340427	TLR6	up	1.50	2.83E-02	1.88E-05
3390121	CASP5	up	1.78	3.28E-02	1.90E-05
7050382	CASP1	up	1.38	3.19E-02	1.90E-05
6100136	CASP4	up	1.56	3.68E-02	1.95E-05
2570291	IFNGR2	up	1.31	3.95E-02	1.97E-05
2470358	IFNGR1	up	1.37	4.61E-02	2.02E-05
3780047	GBP6	up	1.99	5.33E-02	2.07E-05
1980524	GBP4	up	1.47	5.30E-02	2.07E-05
1050020	NLRC4	up	1.48	5.80E-02	2.11E-05
6420750	TLR2	up	1.63	6.67E-02	2.20E-05
1500180	TLR4	up	1.33	1.14E-01	2.73E-05
830440	TLR5	up	1.38	1.31E-01	2.97E-05
1260270	AIM2	up	1.41	1.54E-01	3.32E-05
